# Evaluation of a multi-level intervention to improve postpartum intrauterine device services in Rwanda

**DOI:** 10.12688/gatesopenres.12854.3

**Published:** 2019-02-04

**Authors:** Rosine Ingabire, Julien Nyombayire, Alexandra Hoagland, Vanessa Da Costa, Amelia Mazzei, Lisa Haddad, Rachel Parker, Robertine Sinabamenye, Jeannine Mukamuyango, Julie Smith, Victoria Umutoni, Ellen Mork, Susan Allen, Etienne Karita, Kristin M. Wall

**Affiliations:** 1 Projet San Francisco, Pathology & Laboratory Medicine, Emory University, Kigali, Rwanda; 2 Pathology & Laboratory Medicine, Emory University, Atlanta, GA, 30322, USA; 3 Gynecology and Obstetrics, Emory University, Atlanta, GA, 30322, USA; 4 Health Policy and Management, University of Michigan, Ann Arbor, MI, 48109, USA; 5 Epidemiology, Emory University, Atlanta, GA, 30322, USA

**Keywords:** Post-partum, contraception, birth spacing, family planning, intrauterine device, Rwanda

## Abstract

**Background. **The copper intrauterine device is one of the most safe, effective, and cost-effective methods for preventing unintended pregnancy. It can be used postpartum irrespective of breastfeeding to improve birth spacing and reduce unintended pregnancy and maternal-child mortality. However, this method remains highly underutilized.

**Methods. **We developed a multi-level intervention to increase uptake of the postpartum intrauterine device (PPIUD, defined as insertion up to six weeks post-delivery) in Kigali, Rwanda. High-volume hospitals and health centers were selected for implementation of PPIUD counseling and service delivery. Formative work informed development of a PPIUD counseling flipchart to be delivered during antenatal care, labor and delivery, infant vaccination visits, or in the community. Two-day didactic counseling, insertion/removal, and follow-up trainings were provided to labor and delivery and family planning nurses followed by a mentored practicum certification process. Counseling data were collected in government clinic logbooks. Insertions and follow-up data were collected in logbooks created for the implementation. Data were collected by trained government clinic staff and abstracted/managed by study staff. Stakeholders were involved from intervention development through dissemination of results.

**Results. **Two hospitals (and their two associated health centers) and two additional health centers were selected. In 6-months prior to our intervention, 7.7 PPIUDs/month were inserted on average at the selected facilities. From August 2017-July 2018, we trained 83 counselors and 39 providers to provide PPIUD services. N=9,020 women received one-on-one PPIUD counseling after expressing interest in family planning who later delivered at a selected health facility. Of those, n=2,575 had PPIUDs inserted (average of 214.6 insertions/month), a 29% uptake. Most PPIUDs (62%) were inserted within 10 minutes of delivery of the placenta.

**Conclusions.** This successful, comprehensive intervention has the potential to make a significant impact on PPIUD uptake in Rwanda. The intervention is scalable and adaptable to other sub-Saharan African countries.

## Introduction

The World Health Organization (WHO) recommends postpartum family planning as safe, effective, and cost-effective for prevention of unintended pregnancy, prevention of abortion, birth spacing, and improvement of maternal and newborn health ^1, 2^. Like many sub-Saharan African countries, Rwanda is committed to reducing unmet family planning need, particularly in postpartum women ^3, 4^.

The 2015 Rwandan Demographic and Health Survey (DHS) estimated that 19% of women have an unmet need for family planning ^5^ and the 2010 Rwandan DHS estimated that 51% of women had an unmet need for postpartum (within two years of delivery) family planning ^6^.

In much of the developing world, women with limited access to medical care are often able to attend antenatal care (ANC), labor and delivery (L&D), and infant vaccination services making these visits unique opportunities to address postpartum family planning needs ^7, 8^. Given these multiple entry points, postpartum family planning should not be viewed or offered as a vertical program, but rather as a program integrated into these existing venues for family planning and maternal child health ^9^.

In particular, the copper intrauterine device (IUD) is highly-effective, cost-effective, and can be used immediately postpartum or after 4 weeks postpartum regardless of breastfeeding ^10, 11^. However, the postpartum IUD (PPIUD) remains extremely underutilized across sub-Saharan Africa, including in Rwanda ^11^. The Rwandan Ministry of Health (MOH) previously supported efforts to implement PPIUD services in four district hospitals and eight health centers and found that clinic staff successfully incorporated new skills into ANC and maternity services, inserting 478 PPIUDs over 15 months ^12^. As a result, the Rwandan MOH developed training curricula and reporting mechanisms, and PPIUD is part of the Government’s Family Planning 2020 Commitment ^13^.

However, despite capacity building and interest, uptake of PPIUD services in Rwanda remains extremely low, and overall, the IUD only comprises 2.5% of the method mix among contracepting women between the ages of 15–49 in the 2015 Rwandan DHS (see Family Planning 2020 site).

This low IUD uptake is hypothesized to be due to lack of stakeholders promoting the service, low provider motivation and comfort with the IUD, lack of optimized operational procedures, the often overlooked role of male involvement, and lack of demand-creation strategies informed by clients’ needs and preferences ^12, 14– 17^. Research particularly highlights the need to create demand through providing comprehensive information on contraceptive methods to increase knowledge about benefits and side effects, address misconceptions, and discuss family planning desires with women and couples ^18– 20^. For example, PPIUD uptake has been associated with women’s perceived pregnancy risk, misunderstandings about when women regain fertility after pregnancy, misconceptions about the eligibility requirements to begin family planning methods after delivery ^18^, religious traditions, male involvement, and fear of side effects ^21, 22^. Educational and demand creation efforts are particularly important for the IUD which is less well-known versus other modern methods in sub-Saharan Africa ^23– 26^.

Our objective was to develop and pilot test an evidence-based, multi-level intervention targeting supply, demand, and sustainability to increase uptake of the PPIUD (defined here as uptake up to six weeks after delivery) in Kigali, the capital of Rwanda. Our primary aims were to increase the number of: workers trained to promote the PPIUD to couples/clients in health facilities and the community, providers trained and certified to insert and remove PPIUDs, couples/clients receiving PPIUD educational counseling, and women receiving a PPIUD up to six weeks after delivery. This study was conducted by researchers at Projet San Francisco (PSF).

## Methods

### Ethical considerations and consent

The Emory University Institutional Review Board (IRB) and the Rwanda National Ethic Committee (RNEC) approved the research component (focus group discussions and surveys) of the project (IRB 00001497). Written informed consent was obtained from all participants prior to enrollment. The Emory University IRB determined the programmatic service delivery component of the project (PPIUD counseling and insertions provided by government clinic staff) was exempt from review.

### Intervention framework

**Figure 1.**
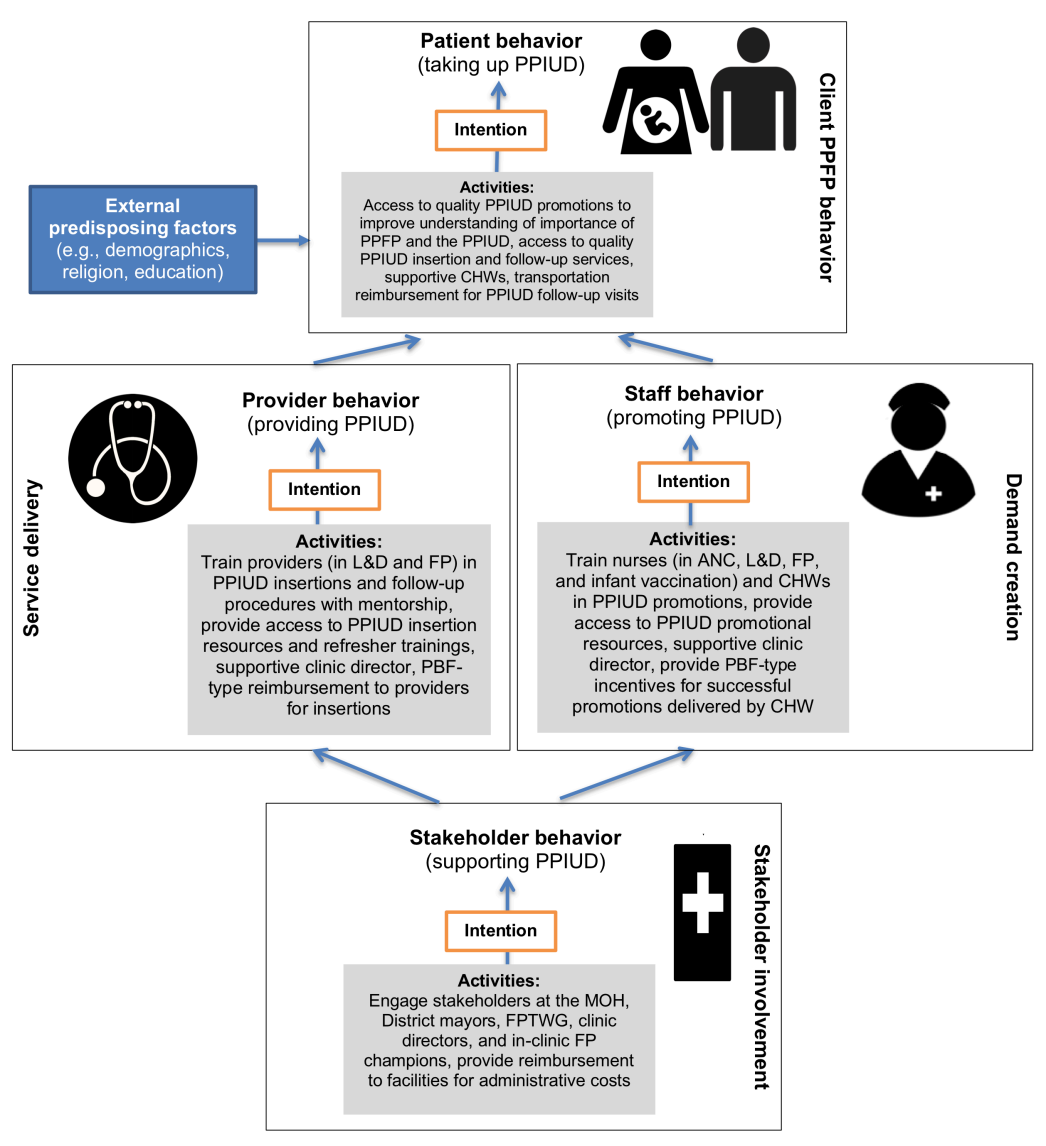
Framework of the PPIUD intervention at multiple levels of the healthcare system. Grey boxes indicate intervention activities designed to change attitudes, norms, and perceived control. PPIUD: postpartum intrauterine device; PPFP: postpartum family planning; FPTWG: Family Planning Technical Working Group; ANC: antenatal care; L&D: labor and delivery; FP: family planning; CHW: community health worker; PBF: performance-based financing; MOH: Ministry of Health.

To develop an evidence-based, multi-level intervention to improve PPIUD supply and demand coordination, our innovative strategy combined behavioral science and operations research methods, specifically using a multi-level implementation science framework based on Greenhalgh *et al*. ^27^ and the Theory of Planned Behavior ^28^. Drawing on input from stakeholders, providers, community health workers, and couple/clients, we designed the intervention to address barriers at multiple-levels. This framework is outlined in Figure 1 and indicates intervention activities designed to change an agent’s attitudes, norms, and perceived control, which in turn affect their intention to either support, provide, promote, or take up a PPIUD. In Figure 1, supply-related activities are described under ‘Service delivery’, demand-related activities are described under ‘Demand creation’, and sustainability-related actives are described under ‘Stakeholder involvement’. These activities are explained in detail below.

### Early stakeholder involvement

Throughout the intervention we received logistical and technical support from a collaborative group of stakeholders at community, facility, non-governmental, and governmental levels. This included the Rwanda MOH, the District Mayors, the Rwandan Family Planning Technical Working Group (FPTWG, which includes governmental stakeholders and non-governmental organization and family planning implementing organizations), and clinic directors and nurse-administrators at the selected hospitals and health centers. Stakeholder support included loan of ‘Mama-U’ (Laerdal Medical) postpartum uterus models for provider trainings by the MOH; engagement in conversations about PPIUD service reimbursement plans (described below) by the MOH, FPTWG, and clinic directors and nurse-administrators; support for facility directors to facilitate PPIUD implementation activities by the MOH and District Mayors.

### Health facility selection and needs assessments

In May-June 2017, a PSF nurse counselor (co-author RS) and a study physician (co-author RI) reviewed government monthly reports from Kigali health facilities to select the highest L&D-volume hospitals and health centers. The number of sites selected were based on budget constraints, and we similarly focused on Kigali only for this pilot study for budgetary and logistical reasons. Two hospitals (and their two associated health centers) and two additional health centers were selected. A PSF nurse counselor (co-author RS) and a study physician (co-author RI) then assessed infrastructure, staff trained in long-acting reversible contraception (IUD or implant) insertions, staff in L&D and family planning, and staff interested as potential trainees for PPIUD services in the selected health facility. They reviewed IUD stocks and any procedures (i.e., PPIUD counseling tools available for use or any PPIUD data collection systems in place) supporting PPIUD supply or demand.

### PPIUD demand creation development

Through formative work in May-July 2017, PSF staff evaluated knowledge, attitudes, and practices regarding PPIUD services among community health workers (CHW) and providers at two high volume health centers which were not selected for our intervention (unpublished manuscript under review; Da Costa V, Ingabire R, Sinabamenye R, Karita E, Umutoni V, Hoagland A, Allen S, Mork E, Parker R, Mukamuyango J, Haddad L, Nyombayire J, Wall KM. An exploratory analysis of factors associated with interest in postpartum intrauterine device (IUD) uptake among pregnant women and couples in Kigali, Rwanda. Submitted 2018; unpublished manuscript under review; Da Costa V, Ingabire R, Sinabamenye R, Karita E, Umutoni V, Hoagland A, Allen S, Mork E, Parker R, Mukamuyango J, Haddad L, Nyombayire J, Wall KM. Perceptions of the postpartum intrauterine device (PPIUD) and implant among pregnant women and couples in Kigali, Rwanda. Submitted 2018). As Rwanda uses a performance-based financing (PBF) system, we also explored provider knowledge of the current PBF structure and other insurance programs for family planning method provision, and asked whether these influenced the methods they provide. We also evaluated knowledge, attitudes, and practices among pregnant women and couples during early ANC visits (men often attend these visits with their partners). Survey and focus group topics included: demographics; previous pregnancy, birth spacing, and family planning history; postpartum long-acting reversible contraception knowledge, attitudes, and practices; and personal and community perceptions of long-acting reversible contraception. Each survey and focus group discussion took approximately 45 minutes to complete, and each individual study participant was compensated $3.60 United States Dollars (USD). We combined information gathered from this formative work as well as a review of existing PPIUD promotional materials ( https://www.k4health.org/toolkits/ppfp/client-materials) to develop our PPIUD counseling flipchart ( Supplementary File 1).

### Postpartum IUD service delivery training

In August 2017, health care providers (nurses and midwives working in L&D and family planning) from our selected government health facilities were trained by two national PPIUD trainers located at the selected district hospitals. Selection criteria for provider trainees were having experience working in L&D or conducting interval IUD insertions in family planning. The training included a 2-day didactic session conducted at PSF (adapted from didactic and practical training materials developed by Jhpiego and USAID in collaboration with the Rwandan MOH).

The timeframe we consider as ‘postpartum’ in this pilot was up to 6 weeks post-delivery (in other work the postpartum time period includes up to one year after childbirth ^9^ to align with the first scheduled infant vaccination visit. This requires insertions at timings which necessitate different skills training (e.g., post-placental and post-partum insertion skills are different from 4–6 week insertions). Didactic and practical trainings focused on all possible insertion timing.

We trained a maximum of 12 trainees per didactic session. The didactic training included information about IUD and PPIUD insertion and removal procedures and follow-up, the use of the PPIUD flipchart in counseling, mock counseling sessions, as well as PPIUD insertion and removal trainings using ‘Mama-U’ (Laerdal Medical) postpartum uterus models. Pre- and post-training tests (adapted from the USAID and Maternal and Child Health Integrated Program Postpartum IUD Training Guide ^29^; Supplementary File 2) consisted of 10 true or false questions and were administered before and after the training.

After passing the didactic training session, two trainees at a time would conduct PPIUD insertions at a selected district hospital under the supervision of a national trainer. The trainees were required to insert correctly and comfortably five PPIUDs under supervision to be PPIUD certified. These five insertions had to include at least one of each of the following PPIUD insertion timings: immediate post-placental, 10 minutes to 48 hours post-delivery, and 4–6-weeks post-delivery. Checklists for PPIUD insertion practices guided the certification process. Intra-cesarean IUD insertions were provided by previously trained doctors who subsequently trained our providers certified in PPIUD.

### Demand creation

In addition to training PPIUD providers to use the PPIUD flipchart, we trained government clinic staff in family planning, ANC, L&D, and infant vaccination to promote the service at the selected facilities. Trainings were comprised of a 3-hour long didactic session led by PSF staff followed by one supervised counseling session.

Based on our previous successful work with CHWs in sensitizing the community about family planning and LARC and referring interested clients to health centers, we trained CHWs from the two hospital-affiliated health centers in charge of pregnant women and newborn health to counsel those women in the community to discuss postpartum family planning, use the flipchart, and encourage women who had received a PPIUD to attend their PPIUD follow-up visits if they missed an appointment. These CHW trainings began in March 2018. CHWs received a 1-day training on the use of the PPIUD flipchart and couples’ family planning counseling strategies. Women were referred to the facility by their CHW if interested to receive an IUD. However, because of the later involvement in the pilot, the role of CHWs was limited and insertions and follow-up appointment resulting from their work are not shown (we recently trained CHW at all the selected facilities in September 2018). CHW were reimbursed for their counseling efforts (see below) and maintained their additional responsibilities which include health assessment of children younger than five, community-based provision of some contraceptives, prevention of non-communicable diseases, and directly observed therapy for tuberculosis. The receive incentives from the government via the community performance-based financing (CPBF) system ^30^.

Thus, women and couples could be provided with PPIUD information at many different time points at the selected health facilities (during ANC, L&D, and infant vaccination up to 6 weeks post-delivery) as well as in the community.

Women still received standard of care family planning counseling, which included counseling on all other family planning methods available. Women were able to choose any method they preferred (or no method). PPIUD counseling was specifically provided as most women have not heard of the IUD and do not know it is available ^23– 26^.

### Implementation procedures

Trainings for providers and counselors were first rolled-out in the hospitals in L&D and their affiliated health centers at the last ANC visits during the initial PPIUD training/certification process. We began our PPIUD counseling sessions during last ANC visits and L&D for higher yield of potential clients. After we had enough clients to ensure that certifications were well underway, we expanded to all ANC visits at the four health centers and L&D in the two non-hospital affiliated health centers. Finally, counselor trainings among providers of infant vaccination services in the health centers were rolled-out. Limited community promotions began near the end of the pilot as described.

We limited our PPIUD service provision to 6 weeks postpartum to correspond with the first infant vaccination visit which is another opportunity to provide PPIUD counseling, service provision, or follow-up visitation. For women counseled during ANC who expressed interest in a PPUID (which was noted on their ANC card), interest was reconfirmed during L&D but women were not counseled again using the flipchart. A PPIUD counseling occurrence was thus recorded if women received the full one-on-one counseling with the flipchart from a trained PPIUD counselor.

### Follow-up procedures

PPIUD follow-up appointments were scheduled 10 days after IUD insertion. Those who had insertions within 48 hours of delivery and who missed their 10-day follow-up visit were assessed at their infant’s first vaccination visit 6 weeks postpartum. Women coming for follow-up appointments were asked if they had complaints. Assessment for signs of infection were conducted via self-reported symptoms (e.g, lower abdominal pain, fever, abnormal discharge, bleeding) and bimanual pelvic exam (e.g., assessing tenderness or pain when moving the cervix and uterus, purulent or yellow cervical discharge, cervical bleeding, tender pelvic mass). Appropriate antibiotic treatment was prescribed immediately given suspected infection per global standards. IUD string placement was checked via bimanual pelvic exam. IUD strings were trimmed as needed and a pelvic ultrasound was recommended if the strings were not visible during physical exam. Women whose IUDs expulsed or who requested a removal were offered the family planning method of their choice. To increase follow-up, in March 2018 we began providing CHWs with lists of clients in their catchment areas who were pending follow-up to remind those women about their appointments.

### Reimbursements and other compensation

PPIUD trainees were provided with *per diem* and transport reimbursement for the time spent training ($5.79 USD per day for approximately two weeks which included the 2-day didactic training plus the mentored certification process). All other reimbursements began in March 2018. Reimbursements to the selected facilities for administrative costs associated with implementing the PPIUD program were provided at $57 USD per month. The facility reimbursement was calculated based on activities facilities would need to facilitate, monitor, and supervise the PPIUD program. This included airtime for communications between providers and directors within facilities on PPIUD activities, clinic directors and the Ministry of Health, and PPIUD program coordinators and district pharmacies for continuous IUD commodity monitoring for prevention of stock outs ($17 USD); an allowance for meetings at the health facilities between PPIUD providers, ANC counselors, CHWs, and facility directors ($28 USD); and a transport allowance for regular family planning supply procurement from district pharmacies to prevent stock outs at the health facilities ($12 USD).

Using the PBF system as a guide ^31^, providers were reimbursed $1.20 USD/PPIUD insertion, and these payments were made to their facility and included in addition to their regular PBF pay. For context, providers receive $0.60 USD/new method user regardless of method type in PBF, and the average salary for nurses working in family planning or L&D is $124-364 USD/month, depending on educational level. CHWs were incentivized $0.57 USD per client presenting their referral when requesting a PPIUD. We also began providing transportation reimbursement for women to attend PPIUD follow-up visits ($2.29 USD/client) at site of insertion as follow-up visits were not part of the routine schedule for new mothers.

### Data collection

PPIUD service delivery and counseling began in August 2017. A unique code unlinked to patient identifiers allowed tracking of clients from the community and ANC through L&D and infant vaccination. Since counseling occurred in several settings, counseling given by CHW were tracked using referral slips. Counseling in ANC included a group talk followed by one-on-one counseling for those expressing interest in family planning. Those receiving one-on-one counseling had their method of interest and estimated date of delivery recorded on their ANC card and in the government logbook which was maintained by government clinic staff. Thus, we only collected data from women receiving the one-on-one counseling sessions.

During insertion, self-reported provider perception of ease of insertion, client anxiety during insertion, and client pain during insertion were captured on scales of 1–10 by our trained PPIUD providers. Insertion data was collected in a logbook created for the project based on one that was already in use by a national PPIUD trainer in one of our selected government facilities. These logbooks were maintained by our trained PPIUD providers in the government facility. Client age and parity data were collected as part of standard procedures in government family planning logbooks.

During follow-up, data collected included method expulsion, genital infection, or method failure (i.e., incident pregnancies occurring after insertion), and client satisfaction with the method was captured on a scale of 1–10. Follow-up data was collected in a logbook created for the project based on one that was already in use by a national PPIUD trainer in one of the selected government facilities. This logbook was maintained by our trained PPIUD providers in the government facility.

Data was extracted and cleaned for data entry into tablets weekly by the PSF field team through the mobile data collection platform Survey CTO v2.41 (Dobility, Cambridge, USA) and uploaded into a Microsoft Access database.

### Analyses

**Figure 2.**
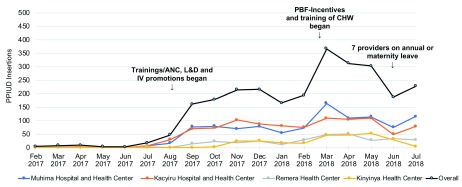
PPIUD insertions over time by facility (N = 2,575 total PPIUD insertions). Percent increase in monthly insertions comparing February 2017-July 2017 to August 2017-July 2018: 2,687%. PPIUD: postpartum intrauterine device; CHW: community health worker; PBF: performance-based financing; ANC: antenatal care; L&D: labor and delivery; IV: infant vaccination.

**Figure 3.**
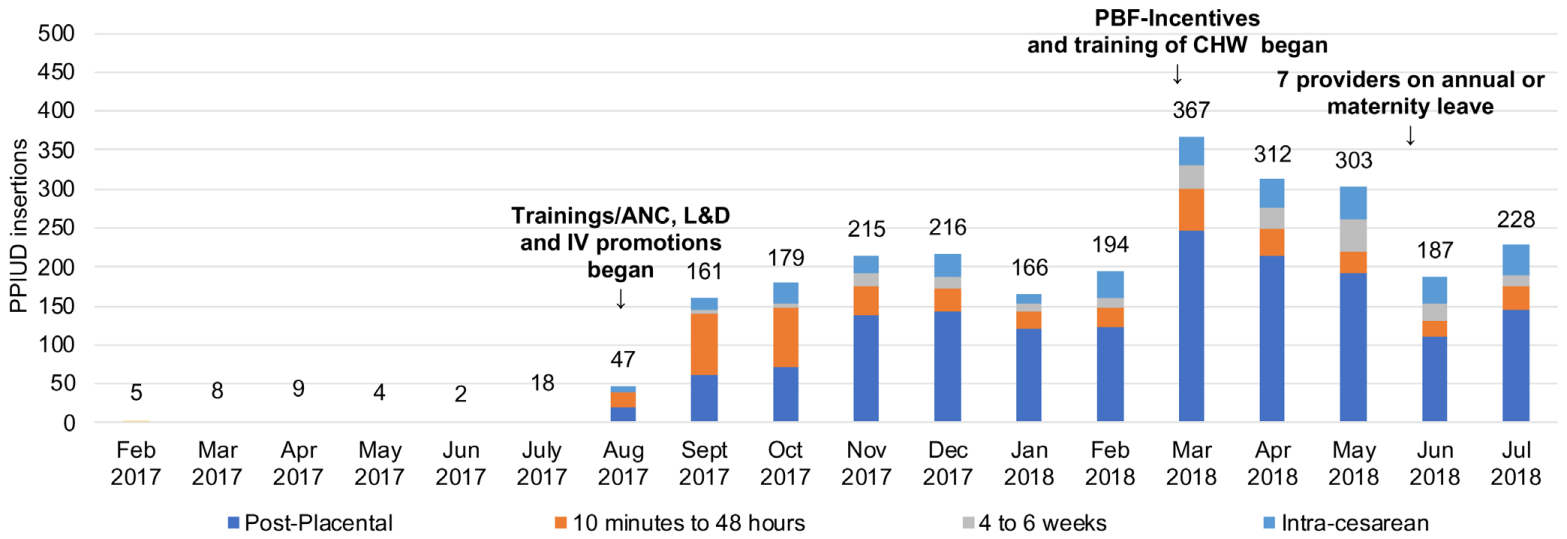
PPIUD Insertions over time by insertion timing (N = 2,575 total PPIUD insertions). PPIUD: postpartum intrauterine device; CHW: community health worker; PBF: performance-based financing; ANC: antenatal care; L&D: labor and delivery; IV: infant vaccination.

Analyses were performed using SAS version 9.4 (Cary, NC). We tabulated, by facility: number of providers trained and certified; number of promoters trained; number of clients who received a one-on-one counseling in a health facility after expressing interest in family planning during group counseling; total number of PPIUDs inserted (overall and by timing of insertion); and number of follow-up visits. From these data, we calculated the proportion of PPIUD uptake among women who delivered at one of our selected facilities and the proportion of insertions by insertion timing; the denominator for this calculation is women who received one-on-one counseling after expressing interest in family planning during group counseling ( Table 1). We also plotted PPIUD uptake over time by facility ( Figure 2) and by timing of insertion ( Figure 3), both after implementation of the intervention and in the six months prior. During these two timeframes, we also calculated the average number of insertions per month. We used descriptive statistics to describe insertion and follow-up data ( Table 2). Two statistical tests were performed to assess the association between timing of counseling and PPIUD uptake (Chi-square test) and the association between the number of counseling sessions received and PPIUD uptake (Chi-square test for trend).

**Table 1.** PPIUD demand creation and service delivery outcomes (August 2017–July 2018).

	Muhima Hospital and Health Center		Kacyiru Hospital and Health Center		Remera Health Center		Kinyinya Health Center		Total	
	N	%	N	%	N	%	N	%	N	%
Deliveries	6369		6712		713		929		14723	
Pregnant women counseled	3758		2635		2025		2089		10507	
Women counseled who delivered in a study L&D ward *	3245	84%	2523	99%	1540	76%	1692	81%	9020	86%
Counseling delivered during:										
Antenatal care	*401*	*12%*	*20*	*1%*	*585*	*38%*	*780*	*46%*	*1786*	*20%*
L&D	*2737*	*84%*	*2523*	*99%*	*332*	*22%*	*216*	*13%*	*5808*	*64%*
Postpartum	*1*	*0%*	*0*	*0%*	*254*	*16%*	*296*	*17%*	*551*	*6%*
Infant vaccination visit	*106*	*3%*	*0*	*0%*	*369*	*24%*	*400*	*24%*	*875*	*10%*
Total number of PPIUD inserted	1028	32%	969	38%	310	20%	268	16%	2575	29%
Post-placental	*513*	*50%*	*744*	*77%*	*197*	*64%*	*136*	*51%*	*1590*	*62%*
Intra-cesarean	*189*	*18%*	*148*	*15%*	*0*	*0%*	*0*	*0%*	*337*	*13%*
10 minutes to 48 hours	*268*	*26%*	*55*	*6%*	*79*	*25%*	*47*	*18%*	*449*	*17%*
4 to 6 weeks	*58*	*6%*	*22*	*2%*	*34*	*11%*	*85*	*32%*	*199*	*8%*

PPIUD: postpartum intrauterine device; L&D: labor and delivery *Denominator for total PPIUD uptake proportions

**Table 2.** Insertion outcomes among women receiving a PPIUD (August 2017–July 2018).

	Muhima Hospital and Health Center		Kacyiru Hospital and Health Center		Remera Health Center		Kinyinya Health Center		Total	
	Mean/N	SD/%	Mean/N	SD/%	Mean/N	SD/%	Mean/N	SD/%	Mean/N	SD/%
Among women receiving a PPIUD insertion (N = 2,575)										
Age (mean, SD)	28.2	6.4	28.8	5.6	28.1	5.5	27.8	6.2	28.3	6.0
Parity (mean, SD)	2.4	1.4	2.4	1.5	2.6	1.4	2.5	1.4	2.4	1.4
Provider perception: ease of insertion (mean, SD) *	9.4	0.7	8.5	1.1	9.7	1.0	9.7	0.6	9.2	1.0
Patient perception: anxiety during insertion (mean, SD) *	1.1	0.4	2.2	0.9	3.9	2.1	1.1	0.6	1.8	1.3
Patient perception: pain during insertion (mean, SD) *	1.1	0.4	2.5	0.9	3.6	2.0	1.0	0.2	1.9	1.3
Among attending PPIUD follow-up (N = 1,399)										
Expulsion (N, %)										
Yes	35	7%	28	5%	6	3%	8	5%	77	6%
* IUD reinserted*	*26*	*74%*	*10*	*36%*	*3*	*50%*	*7*	*88%*	*46*	*60%*
* Implant inserted*	*2*	*6%*	*5*	*18%*	*1*	*17%*	*1*	*13%*	*9*	*12%*
* No LARC inserted*	*7*	*20%*	*13*	*46%*	*2*	*33%*	*0*	*0%*	*22*	*29%*
No	457	93%	506	95%	191	97%	168	95%	1322	94%
Infection (N, %)										
Yes	5	1%	1	0%	0	0%	0	0%	6	0.4%
No	487	99%	531	100%	197	100%	176	100%	1391	99.6%
Failure (N, %)										
Yes	0	0%	0	0%	0	0%	0	0%	0	0%
No	492	100%	534	100%	197	100%	176	100%	1399	100%
Removal (N, %)										
Yes	7	1%	5	1%	4	2%	3	2%	19	1%
No	484	99%	528	99%	193	98%	173	98%	1378	99%
Patient report of satisfaction with PPIUD (mean, SD) *	9.9	0.6	9.9	0.5	10.0	0.2	9.9	0.4	9.9	0.5

*From a score of 1–10 SD: standard deviation; PPIUD: postpartum intrauterine device; LARC: long-acting reversible contraception

## Results

### Health facility selection and needs assessments

The health facilities selected included Muhima and Kacyiru hospitals (and their associated health centers) and Remera and Kinyinya health centers. The two hospitals, Muhima and Kacyiru, provide routine L&D services for their adjoining health centers and also receive referrals of high-risk and complex obstetric cases from several other health centers. Muhima and Kacyiru health centers provide ANC, family planning, and infant vaccination. The other two health centers, Kinyinya and Remera, were distant from the selected hospitals and from each other, and included routine L&D as well as ANC, family planning, and infant vaccination services. Complex and high-risk obstetric cases from these latter two health centers were referred to nearby hospitals not included in this study. All facilities had infrastructure for IUD insertions and procurement but did not have PPIUD insertion kits or Kelley forceps which were procured. Though two of our selected sites participated in PPIUD training interventions (not delivered by our team) several years prior to our pilot implementation, outside of two identified national PPIUD trainers (one located at each selected hospital) no providers stated having been trained on PPIUD insertion and no PPIUD counselors were active at the selected facilities.

### PPIUD demand creation

Four client focus groups comprised of 32 participants and 14 provider interviews informed the development of surveys which were conducted among 14 health providers, 24 CHWs, and 150 women or couples attending ANC visits. Information gathered from this formative work (under review) led to the development of the PPIUD counseling flipchart. Briefly, the themes identified during formative work included lack of information on birth spacing and the IUD and specific lack of information and misconceptions related to the PPIUD. Based on this formative work, the PPIUD flipchart contained information addressing the importance of birth spacing; describing the mechanism, longevity, effectiveness, and cost of the IUD; discussing return to fertility after removal, expulsion rates, and insertion timing options (including post-cesarean-section insertions); overviewing possible side-effects such as pain and heavy bleeding; and dispelling misconceptions (such as that the IUD affects headaches, weight gain, breastfeeding, sexual intercourse, and HIV status, as well as the commonly expressed concern that one is not able to remove the method at any time). Though the focus of the counseling and promotional flipchart was on the PPIUD, the flipchart also included discussion of the implant, which is more well-known and widely available ^24^. The flipchart was designed to be delivered by clinic providers during ANC, L&D, infant vaccination, or by CHWs in the community to both pregnant women and couples ( Supplementary File 1) along with standard of care family planning counseling which included counseling on all other family planning methods available.

### Postpartum IUD demand creation and service delivery outcomes

We trained a total of 83 staff to promote PPIUD to couples/clients in-clinic and in the community. Forty-nine were in-clinic PPIUD counselors while 34 were CHWs (who were engaged in March of 2018). Thirty-nine providers were trained to deliver the service (with pre-test scores averaging 7.5/10, post-test scores averaging 9/10, and no post-test failures), and 90% of those were certified by July 2018. The remaining 10% either moved to different departments (such as tuberculosis or data monitoring and evaluation) within the selected health facilities or took jobs outside of the selected facilities before completing their certifications. Trainees were midwives and nurses working in L&D (85%) or from family planning clinics (15%). The majority of our trainees were women (80%). Because we began our initial PPIUD counseling promotions to women in ANC and L&D, we hypothesized that most insertions would occur in L&D and therefore trained a larger proportion of L&D providers on PPIUD insertions. No trainees had prior PPIUD experience.

From August 2017-July 2018, n=9,020 pregnant women were counseled one-on-one who later delivered at one of the selected facilities (48% of these expressed interest in PPIUD at the time of counseling). Of these women, we were able to link 70% from counseling to insertion to follow-up with unique identifiers, and the remaining women were linked using a combination of ANC, phone, registration, and family planning ID numbers from government clinic logbooks. For context, 14,723 deliveries occurred at the selected facilities during the implementation timeframe.

Most counseling took place during L&D (64%) or ANC (20%). Overall, n=2,575 PPUIDs were inserted (29% PPIUD uptake among women who received one-on-one counseling sessions after expressing interest in family planning during group counseling) ( Table 1). Timing of counseling was associated with uptake (p<0.001), with highest uptake for counseling delivered during L&D (34%) and the lowest uptake for counseling delivered during ANC (9%, Dataset 1 ^32^). Most (95%) women only received one counseling session, with 4% receiving two counseling sessions (and 1% receiving three to four counseling sessions). Increasing number of counseling received was associated with PPIUD uptake (p=0.04, Dataset 1 ^32^).

### PPIUD insertions over time by facility

In the 6-months prior to the intervention (February 2017-July 2017), 46 PPIUDs were inserted in the selected health facilities (average of 7.7 insertions/month). During our 12-month intervention, 2,575 PPIUDs were inserted (average of 214.6 insertions/month). The percent increase comparing monthly PPIUD insertions between February 2017-July 2017 to August 2017-July 2018 was 2,687% ( Figure 2).

We saw an immediate increase in PPIUD uptake as training/counseling began which decreased slightly after initial trainings/supervised certifications ended. Once reimbursements began along with training of CHWs, we again observed an increase in insertions. Insertions decreased in June 2018 as seven PPIUD certified nurses began their annual or maternity leave, followed by a subsequent increase as certified providers rearranged their workloads accordingly to compensate.

### PPIUD insertions over time by insertion timing

In the initial three months of the program, insertions placed between 10 minutes to 48 hours post-delivery were the most common (45% of all insertions), but from November 2017 onward post-placental insertions were the most common (66% of all insertions). Overall, 62% of insertions were immediately post-placental, 17% occurred 10 minutes to 48 hours post-delivery, 8% occurred 4 to 6 weeks post-delivery, and 13% occurred intra-cesarean section ( Figure 3).

### Insertion outcomes among women receiving a PPIUD

Of the 2,575 women receiving PPIUDs, the average age was 28.3 and average parity was 2.4. Provider perception of ease of insertion was high across facilities (average score of 9.2/10), and patient perception of anxiety and pain were low (average scores of 1.8/10 and 1.9/10, respectively) ( Table 2). Remera health center had slightly higher than average patient anxiety and pain scores relative to the other facilities.

N=1,399 (60%) women who were due for PPIUD follow-up visits attended them. Overall proportions of expulsions were low at 6% (N=77), and 60% of women who experienced an expulsion had an IUD reinserted. Expulsion proportions were similar by timing of insertion. Infections were extremely uncommon across all facilities at 0.4%, and no cases of IUD failure were identified. One percent of women requested removals, with the most commonly cited reason for removal being that the husband (32%) or the woman (26%) did not like the method (Dataset 1 ^33^). Overall satisfaction with the PPIUD was very high across all facilities (average score of 9.9/10).

## Discussion

In this PPIUD implementation in government health facilities, we focused on supply, demand, and stakeholder engagement to significantly increase the provision and uptake of the PPIUD. The proportion of women who were made aware of this service and selected this method after delivery was high as was the proportion of insertions that were post-placental. Client satisfaction with the service was high and removal, expulsion, and infection proportions were low.

### Supply

As has been shown in extensive previous work from other groups ^8, 12^, our study demonstrated that it is feasible to train government providers to deliver consistent, quality PPIUD services that are adaptable with their current workloads. Our provider training certification process was very rigorous, requiring insertions at all time points and with structured training and mentoring. Staff turnover and leave was a challenge, and new and refresher trainings will be needed over longer timeframes. However, staff began to train each other near the end of the pilot implementation and took over intra-cesarean section insertions from previously trained doctors, indicating the sustainability of our model. Though we encountered no issues with IUD stockouts, other studies have observed such challenges, and the potential for device stockouts must be monitored.

### PPIUD demand creation and uptake

Provider training and infrastructure alone is not sufficient to ensure the success of PPIUD services and increase demand, especially for the less well-known IUD ^24, 34^, and several demand creation strategies may be needed. A primary contribution of our work is in supporting demand creation using a counseling tool we developed based on extensive formative work and designed to improve client knowledge of postpartum family planning options, discuss the PPIUD in particular including addressing common misconceptions and concerns, and be delivered to both women and their male partners. We observed an increase in PPIUD uptake pre- versus post- intervention after PPIUD supply and demand coordination began.

Most insertions occurred during L&D which is reflective of the fact that we began PPIUD counseling during last ANC visits and L&D for higher yield of potential clients during the PPIUD training/certification process. We found that providing counseling during early (non-active) labor was acceptable as women were often several waiting hours to deliver in common areas at the facilities; in fact, L&D was the only venue for counseling employed by the two national trainers prior to our study. Since most women received counseling during L&D, they were only counseled once. A study in Nigeria showed that repeated postpartum family planning counseling over multiple ANC sessions increased postpartum family planning use ^35^, as we similarly observed an association with multiple counseling sessions.

It is interesting that PPIUD uptake after counseling delivered during ANC was relatively low. It is possible that some women counseled during ANC receive insertions at a later time point (i.e., between delivery and their first infant vaccination visit) at facilities that were not one of our selected facilities. Our inability to track women outside of our selected facilities is a limitation of working in relatively few facilities, and more work needs to be done to explore the low uptake after ANC promotions. 

The role of CHWs in PPIUD counseling was limited to the last few months of this pilot and it is unclear what effect our late introduction of CHW counseling had since we began CHW trainings and reimbursements over a similar timeframe. In future studies, we will expand CHW counseling and conduct comparative effectiveness studies of clinic-based versus community-based counseling strategies.

Importantly, our counseling flipchart also discussed the implant, and we describe implant uptake in a forthcoming analysis.

### PPIUD insertion timing

Most PPIUD insertions were post-placental (60%) and the second most frequent timing of insertion was 10 minutes to 48 hours after delivery indicating that our counseling often led to insertion before women leave the facility after delivery. In a study that integrated PPIUD services into maternal care facilities in six low- and middle-income countries, researchers found that in Rwanda, 27% of PPIUD insertions were post-placental, 43% were intra-cesarean, and 30% were within 10 minutes to 48 hours of delivery; in this study, insertion timings varied widely by country with Rwanda having the lowest proportion of insertions being post-placental ^8^. In a study on providing postpartum family planning services in West and Central Africa, most PPIUD insertions were intra-cesarean (33%) with relatively fewer being post-placental (20%) ^36^.

Though we cannot conclusively state why post-placental insertions became the most popular insertion timing over the course of our pilot, we hypothesize that providers became more comfortable inserting during this time period with more practice, and that post-placental insertions may be easier for both the provider and patient (as women are already positioned for insertion and the cervix is dilated). Further study is needed to explore why post-placental insertions became the most popular insertion timing choice.

We hypothesize that insertion uptake during the 4–6 week period is relatively low since we began our counseling training among ANC and L&D providers and trained infant vaccination providers to provide PPIUD counseling later in the implementation as described. While we did not train our providers on intra-cesarean PPIUD insertions during the formal training process, some were trained by facility doctors after they were certified. This possibly explains the relatively lower intra-cesarean insertion proportion despite the fact that we were working in two large hospitals where cesarean sections were not infrequent. Future studies will incorporate intra-cesarean insertions into our formal training and certification process.

### PPIUD follow-ups

PPIUD follow-up proportions may be affected by women attending other nearby health centers not included in this study for follow-up, or simply not attending follow-up visits. Similarly, in a study in providing postpartum family planning services in West and Central Africa, 42% of women who had a PPIUD inserted also attended follow-up (13.8% in person at the clinic between 2–6 weeks, and 28.6% by phone at 6 weeks) ^36^.

Of those with PPIUD follow-up appointments, reported satisfaction with the method was high, and we observed very few adverse outcomes during the study. PPIUD expulsions were relatively rare (6%). Infections and removals were also rare (<=1% of insertions) and no failures were observed. Similarly, in a study that integrated PPIUD services into maternal care facilities in low- and middle-income countries, expulsion rates were low (ranging from 2–4%), infection rates were low (0–1%), and removals ranged from 1%–11% ^8^. In a study in providing postpartum family planning services in West and Central Africa, 0.8% of PPIUD users self-reported expulsions and only 0.5% (n=12) requested removal (10 desired pregnancy and two had husbands who disapproved of the PPIUD) ^36^. These and our data are reassuring regarding PPIUD insertions and adverse events.

While the majority of follow-ups occurred at the 6-week infant vaccination visit, women were counseled to come for follow-up 10 days after insertion. This was done to try to increase follow-up proportions *at our selected facilities* (as many women may seek care including their first infant vaccinations at a facility closer to them that was not one of our selected facilities). This is an alteration of the Jhpiego protocol which recommends 4–6 week follow up visits given no issues (as the strings may not have descended into the cervix prior to 4–6 weeks). Ongoing and future work in expanded facilities will use the protocol of 6-week follow-up for women who are not having any issues with their insertions.

If no IUD strings were visualized on bimanual pelvic exam, women were given ultrasounds which may not be a sustainable protocol in non-urban settings. The use of the Jhpiego ‘no strings’ algorithm may be more practical in settings without an ultrasound (which includes using a sterile cervical brush or narrow forceps to probe the cervical canal, x-ray, or waiting for a future visit for strings to descend with use of a back-up contraceptive method in the meantime) ^37^

### Male involvement

Given that most of the relatively few PPIUD removals in our study were due to male partner’s not liking the method, male involvement during counseling may be very important. A review of 26 postpartum family planning studies in low- and middle-income countries found that male partner involvement may increase knowledge and use of postpartum contraception ^38^, and other studies found male partner involvement is important for postpartum contraception uptake and continued use ^32, 39– 41^. Men were present for 24% of our PPIUD counseling sessions, and this proportion can likely be increased as we now offer PPIUD counseling on first ANC visits during which men are more likely to attend (in this pilot, we began PPIUD counseling during last ANC visits for higher yield of potential clients during the PPIUD training/certification process, and many men do not attend those). As we expand beyond this pilot, the role of male involvement will be evaluated. We are currently conducting focus groups and surveys to further explore the role of male involvement in postpartum family planning choices.

### PBF-type incentives

PBF-type incentives may have increased the uptake of PPIUDs in our study by offsetting administrative costs incurred by facilities and time costs to providers. Providers were incentivized beyond their PBF in this pilot to provide two methods – the IUD and the implant – because those methods take additional skill and timing to provide. The incentive is thus viewed as additional payment for providers’ work. CHW reimbursements were minimal and future cost analyses may indicate that this is a cost-effective method for maintaining sustainable services.

### Sustainability

The intervention was developed with an eye toward sustainability. A review of 31 publications about promotion of IUDs in low- and middle-income countries found that lack of population impact and sustainability was in large part attributable to the fact that most interventions were not initiated with government support and in government facilities ^42^. To overcome this obstacle, our intervention builds on the substantial past work of groups such as FHI360 and Jhpiego which have shown that delivery of PPIUD services in government clinics in Rwanda is feasible, and we relied heavily on the training curriculum developed from past efforts. Our intervention was designed with key stakeholder and MOH input to operate in government facilities led by trained government staff. We will continue to collaborate with MOH stakeholders to share our findings for ultimate hand-off to the MOH (as was done by our research team with couples’ voluntary HIV counseling and testing ^43^).

Additionally, we are working to expand our understanding of how the Rwandan PBF and government-sponsored health insurance programs influence PPIUD provision. Ensuring there are adequate resources, time, and motivation for providers to focus on PPIUD training, counseling, and insertion is challenging ^44^. Providers cannot provide such services long-term without appropriate support nor can clinic directors support such services without a way to offset costs. While government health insurance programs reimburse a nominally higher amount for IUD insertions versus oral or injectable contraception, they do not reimburse PPIUDs inserted before leaving the facility. Currently these are the majority of PPIUDs inserted in government facilities and are considered a revenue loss since they attract no insurance reimbursement. This affects mostly hospitals as they have high-volume L&D wards. The PBF system, also government-sponsored but focused specifically on reimbursing hospital and health center staff for performance, reimburses providers a flat rate regardless of type of contraception. Thus, the current PBF system may *disincentivize* provision of PPIUDs which requires additional provider time and training. Though there is a theoretical concern that altering these systems could increase provider bias ^44^ this must be weighed against the need to appropriately compensate providers and facilities. We are currently exploring stakeholder and policymaker perceptions related to restructuring the PBF reimbursements for family planning methods based on the skill and time it takes to provide them.

Finally, our model was successful in combining service delivery with demand creation by simultaneously training PPIUD providers and counselors, and these trained providers may be able to support ongoing training. Staff began to train each other near the end of the pilot implementation and took over intra-cesarean section insertions from previously trained doctors. As we develop more PPIUD trainees and counselors, our selected health facilities could serve as training centers for expansion to other facilities in Kigali and rural areas (notably, the MOH has already requested that several of our trainees provide PPIUD service provision training to other facilities in Kigali).

Ongoing studies will be useful to determine whether these components are effective at creating sustainability in the long-term.

### Limitations

Several limitations warrant discussion. Group counseling sessions were often conducted in ANC, L&D, and infant vaccination and time constraints limited the number of women who could receive a subsequent one-on-one counseling session to those who were interested in family planning. As a result, more women heard about the PPIUD than were recorded, and our estimates of PPIUD uptake should not be compared to other studies which use a different estimate of the denominator. The two hospitals had large volume L&D services that included referrals of high-risk and complex cases from non-participating clinics. If those PPIUD clients did not return to one of our selected health facilities for follow-up assessment, they would not be captured. Because we were collecting service delivery data, we do not have extensive demographic information to explore demographic factors associated with uptake. Similarly, we did not collect data on why women selected or did not select the PPIUD. We are currently conducting surveys with women who received our postpartum family planning counseling to explore these reasons. Additionally, we based our counseling flipchart on the formative work and previous experience developing couples’ family planning flipcharts, but the counseling strategy was not based on evidence-based counseling techniques (e.g., Balanced Counseling Strategy) and future incorporation of such evidence-based techniques could be helpful. Since providers assessed their own and client perceptions regarding PPIUD insertions via self-report, this could lead to bias (possibly with provider’s overestimating the ease of insertion and client’s underestimating their pain or anxiety associated with the procedure). Finally, given the pre-post study design, it is not possible to rule out the effect of secular changes on PPIUD uptake, though no national interventions or other similar projects were taking place in the capital during our implementation.

## Conclusion

With renewed interest in postpartum IUD services, this comprehensive multi-level intervention is extremely well-timed and has the potential to make an impact on PPIUD uptake in Rwanda. Lessons learned from this and other PPIUD interventions show the critical and interconnected role of stakeholder support, training with mentored supervision, demand creation, and monitoring and evaluation. We are working with stakeholders to share best practices, and a cost-effectiveness analysis of the intervention is underway. We are planning to expand the service to other hospitals and health centers in Kigali which could become training centers for other facilities. We believe that our PPIUD implementation model, which achieved high PPIUD acceptance with high satisfaction and low adverse effects, is replicable and expandable.

## Consent

The Emory University Institutional Review Board (IRB) and the Rwanda National Ethic Committee (RNEC) approved the research component (focus group discussions and surveys) of the project (IRB 00001497). Written informed consent was obtained from all participants prior to enrollment. The Emory University IRB determined the programmatic service delivery component of the project (PPIUD counseling and insertions) was exempt from review.

## Data availability

Underlying data is available from Harvard Dataverse. Dataset 1: Replication Data for: an interim evaluation of a multi-level intervention to improve postpartum intrauterine device (PPIUD) services in Rwanda https://doi.org/10.7910/DVN/WLZ7PC ^33^

Data is available under a Creative Commons Zero (“CC0”) Public Domain Dedication Waiver

## Supplementary material

Supplementary File 1: Postpartum intrauterine device educational and promotional flipchart.

Supplementary File 2: Pre- and post-training test.
