# Supplementary material for: Evaluation of a multi-level intervention to improve postpartum intrauterine device services in Rwanda
Source: Gates Open Res. 2019 Feb 4;2:38. Originally published 2018 Aug 20. [Version 3] doi: 10.12688/gatesopenres.12854.3 (PMC6266741; doi:10.12688/gatesopenres.12854.3)
Supplement: Supplementary file 1 [file gatesopenres-2-14022-s0000.tgz › c42d8e9e-6242-4480-9709-cfdda42c037e.pptx]

## Slide 1
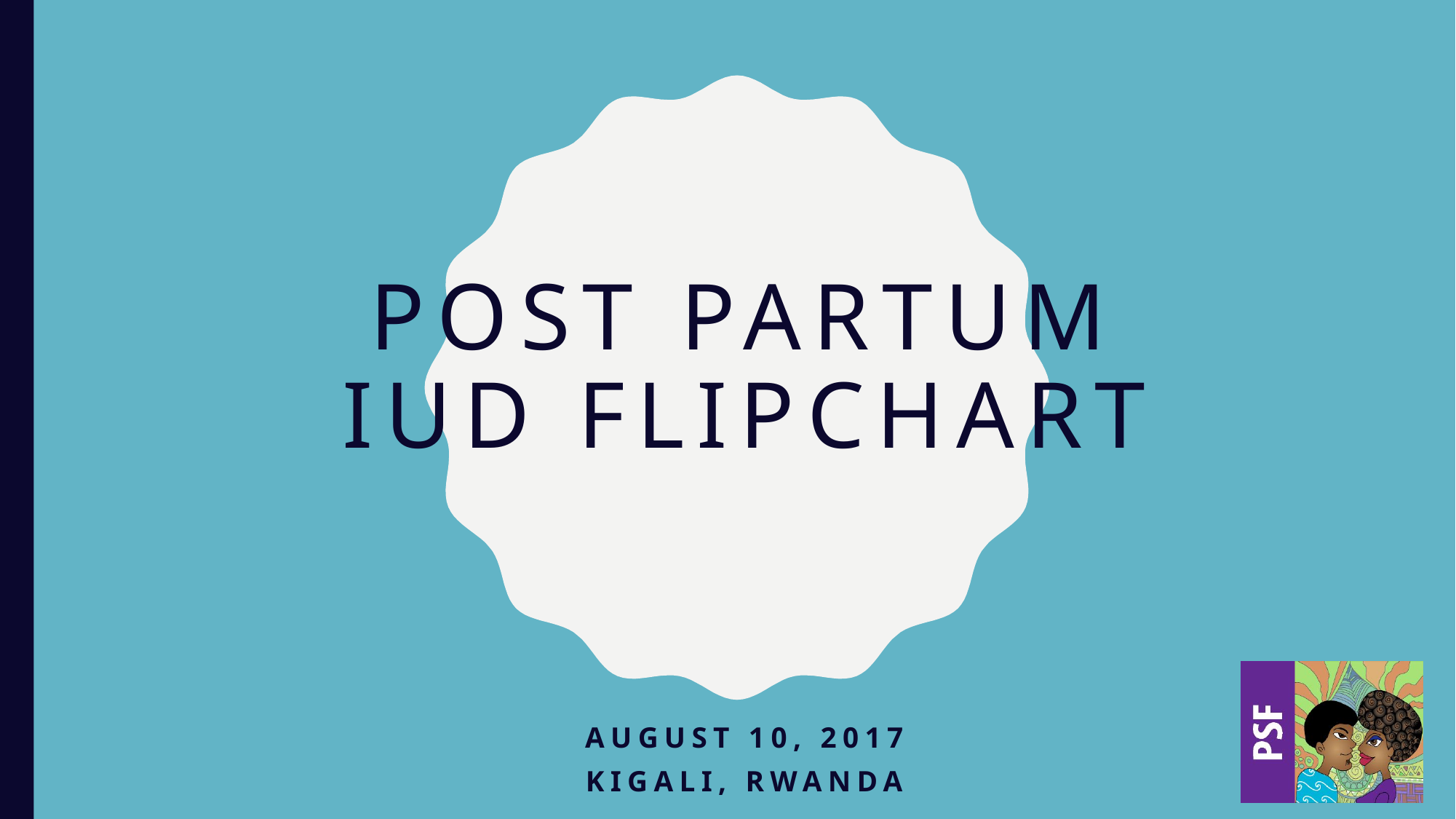

# Post partum IUD flipchart
August 10, 2017
Kigali, Rwanda

## Slide 2
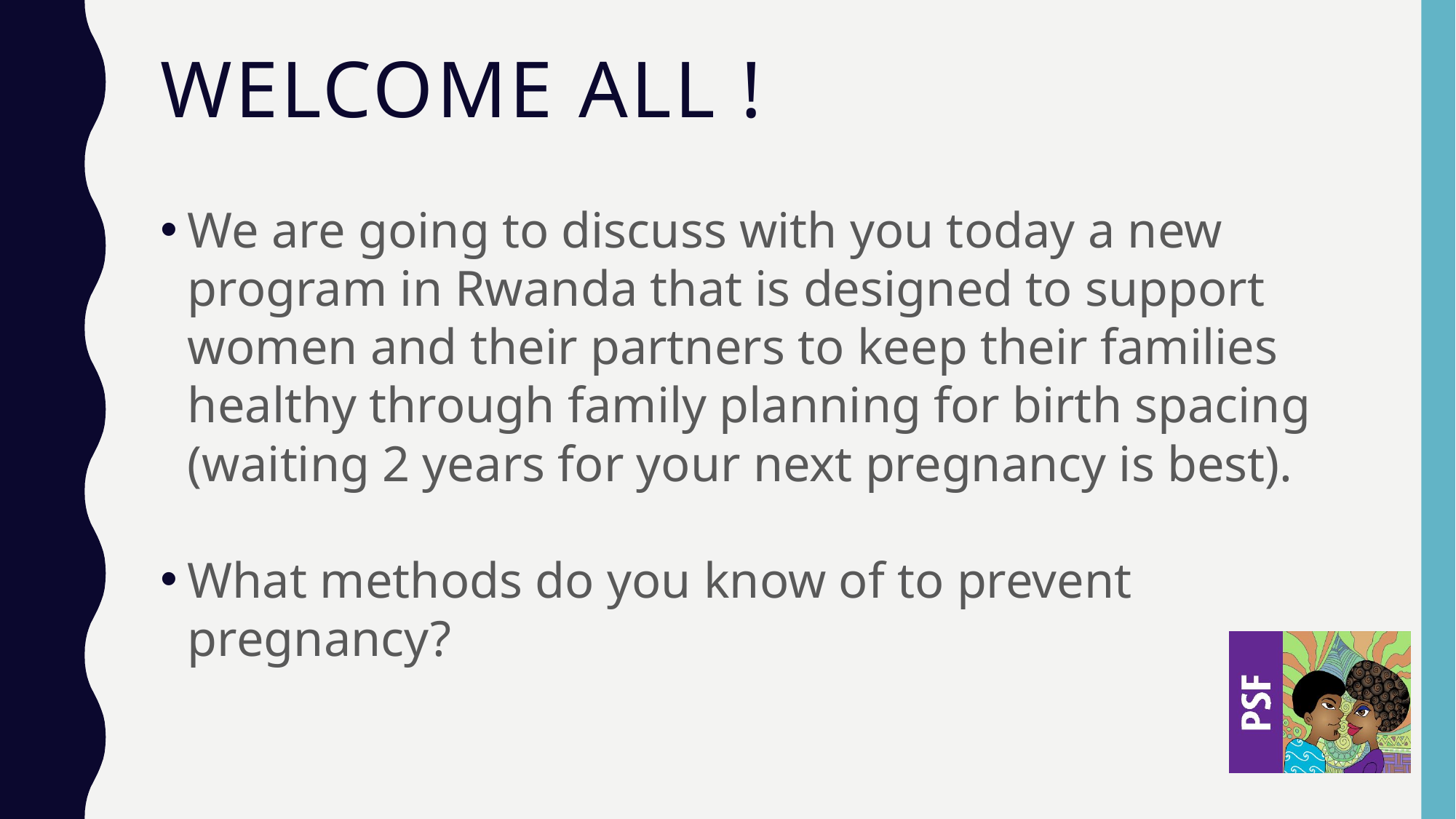

# Welcome all !
We are going to discuss with you today a new program in Rwanda that is designed to support women and their partners to keep their families healthy through family planning for birth spacing (waiting 2 years for your next pregnancy is best).
What methods do you know of to prevent pregnancy?

## Slide 3
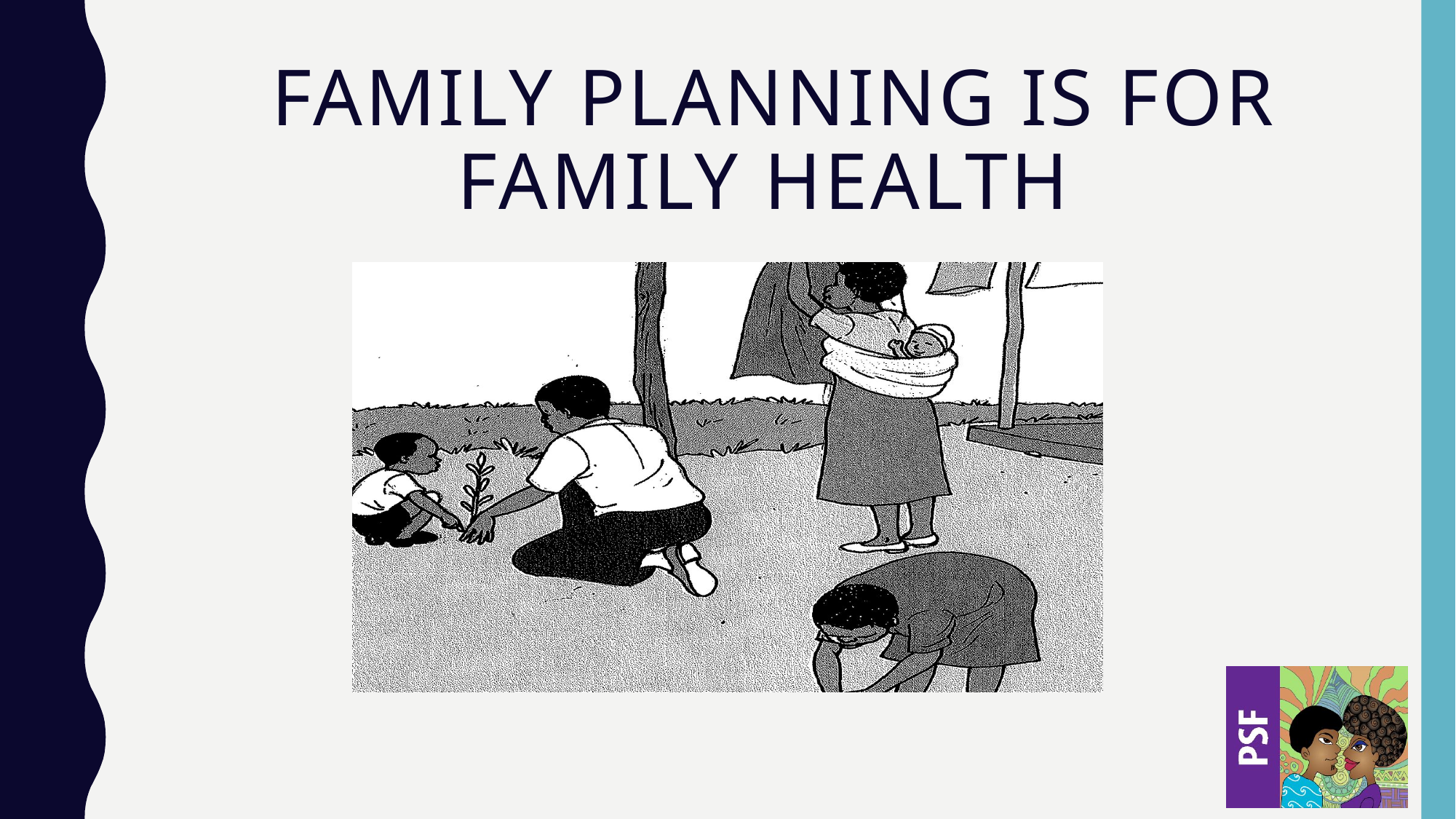

# Family Planning is for Family health

## Slide 4
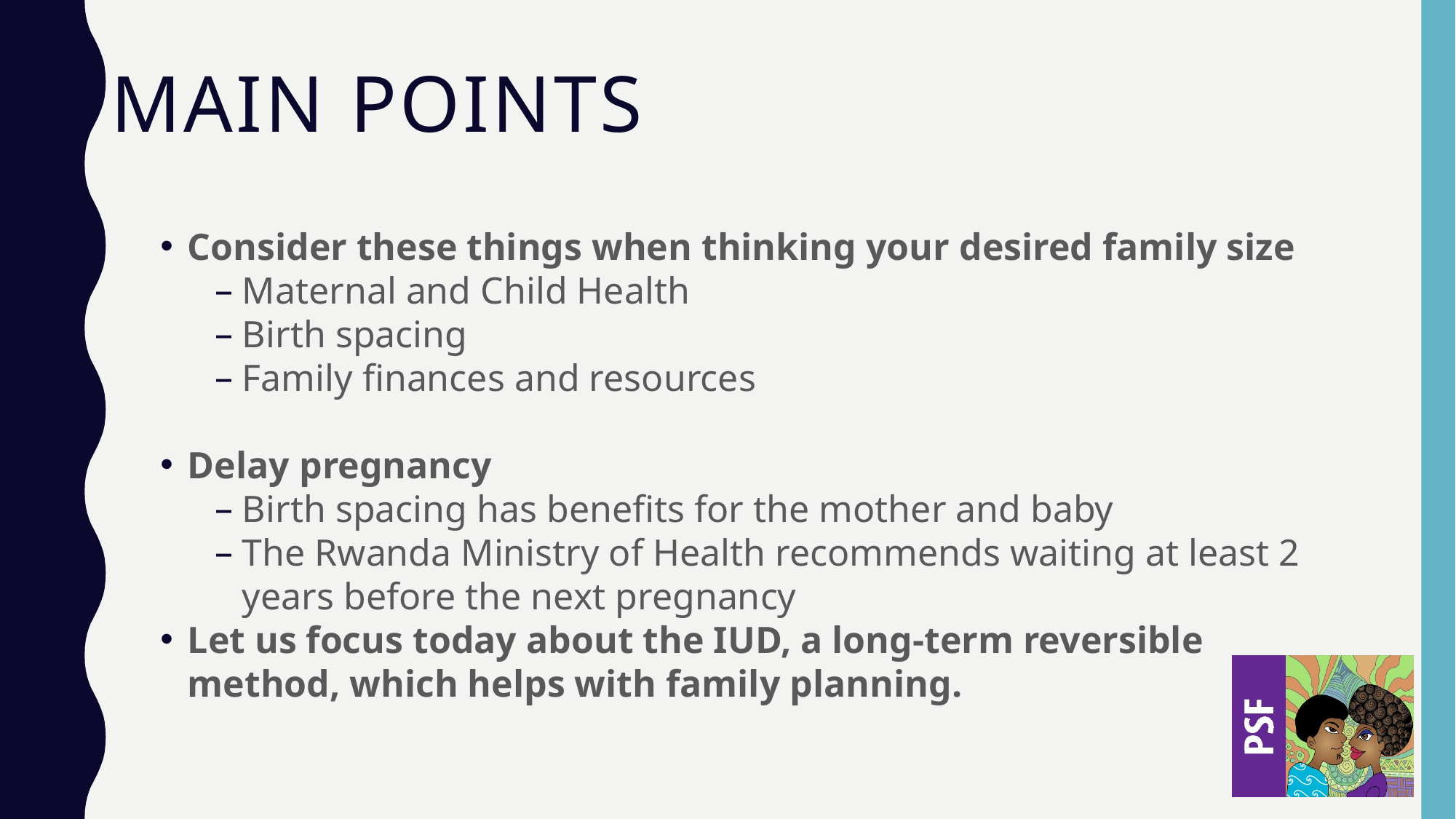

# Main points
Consider these things when thinking your desired family size
Maternal and Child Health
Birth spacing
Family finances and resources
Delay pregnancy
Birth spacing has benefits for the mother and baby
The Rwanda Ministry of Health recommends waiting at least 2 years before the next pregnancy
Let us focus today about the IUD, a long-term reversible method, which helps with family planning.

## Slide 5
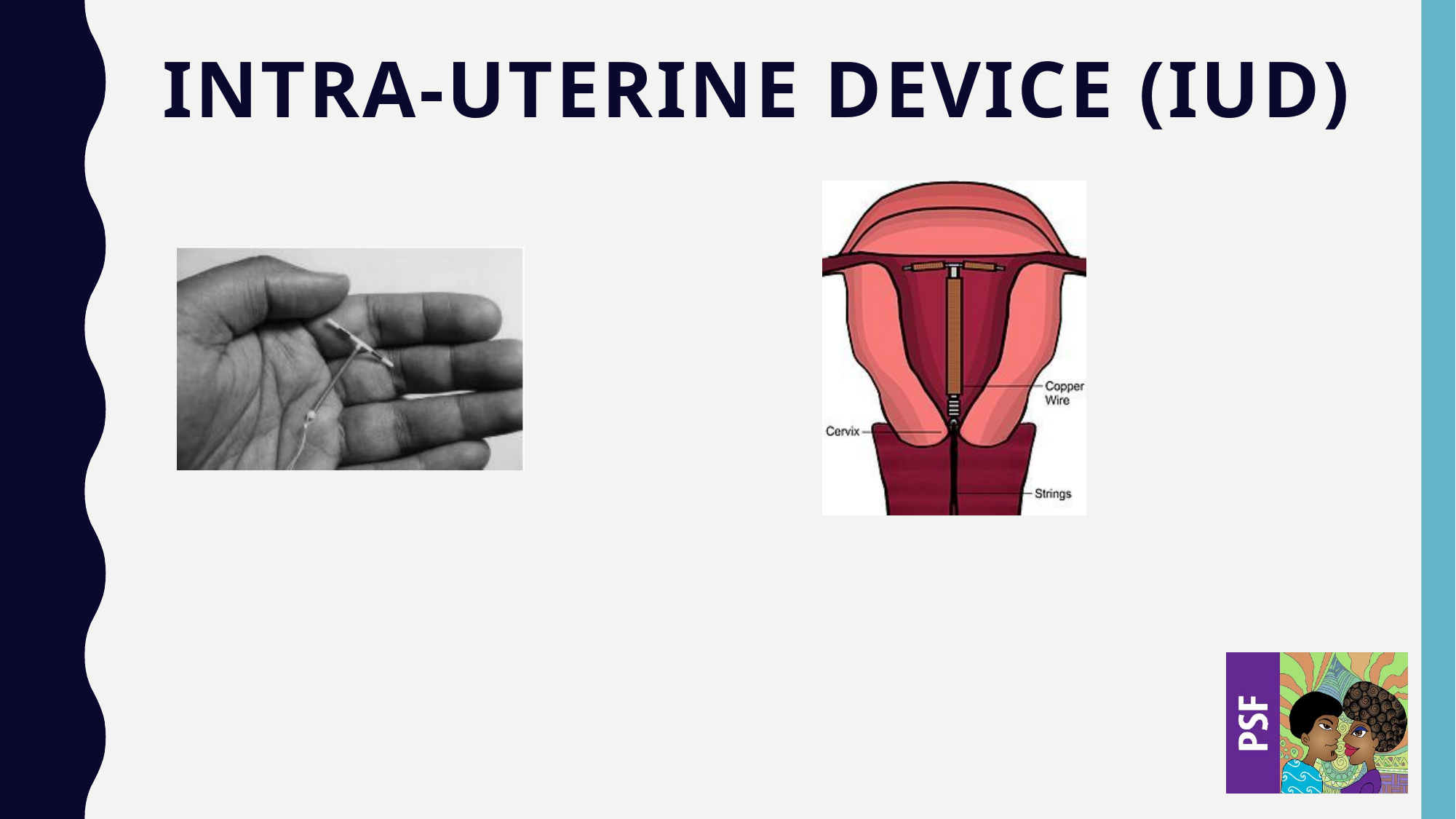

# Intra-Uterine Device (IUD)

## Slide 6
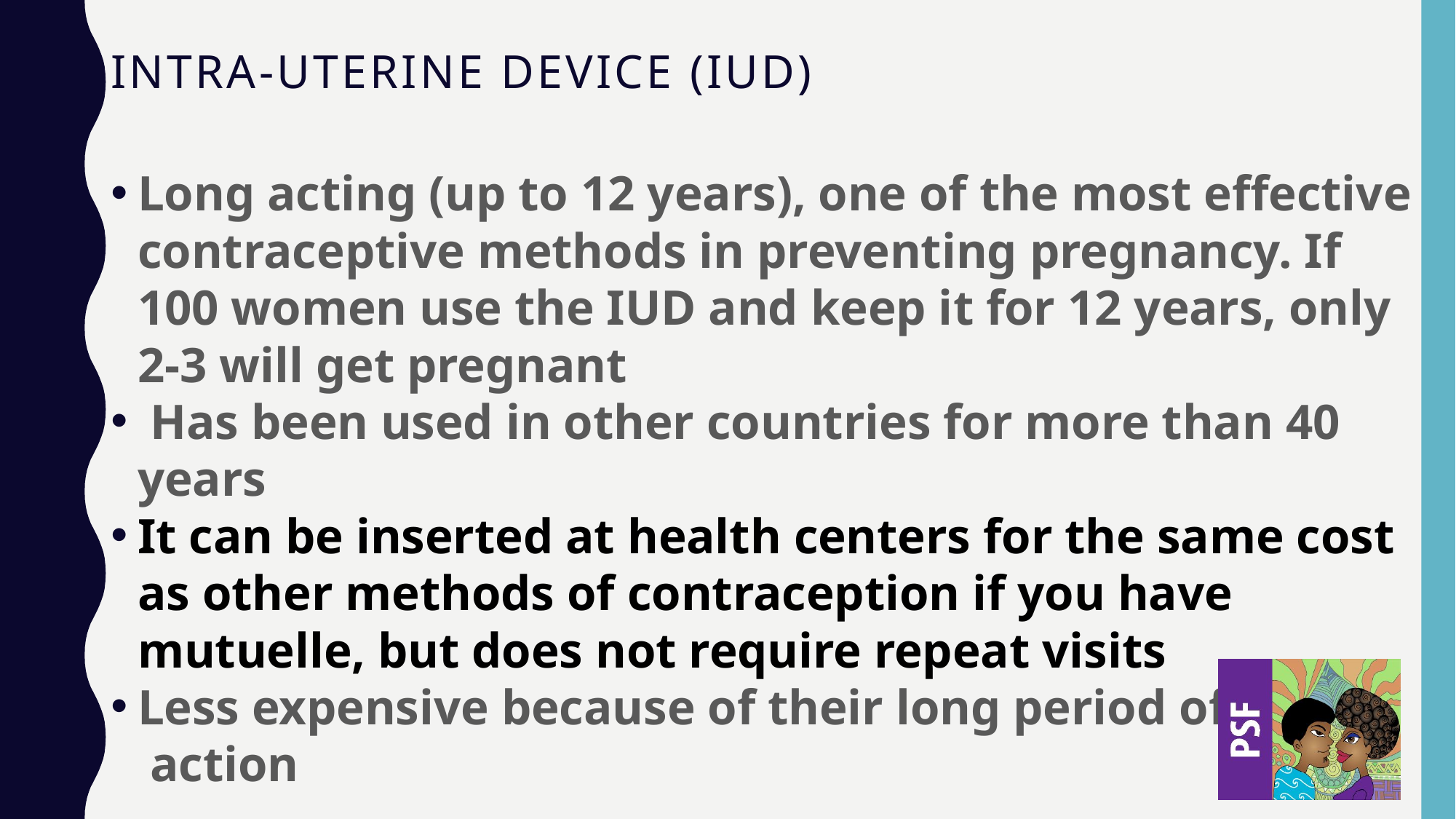

# Intra-Uterine Device (IUD)
Long acting (up to 12 years), one of the most effective contraceptive methods in preventing pregnancy. If 100 women use the IUD and keep it for 12 years, only 2-3 will get pregnant
 Has been used in other countries for more than 40 years
It can be inserted at health centers for the same cost as other methods of contraception if you have mutuelle, but does not require repeat visits
Less expensive because of their long period of action

## Slide 7
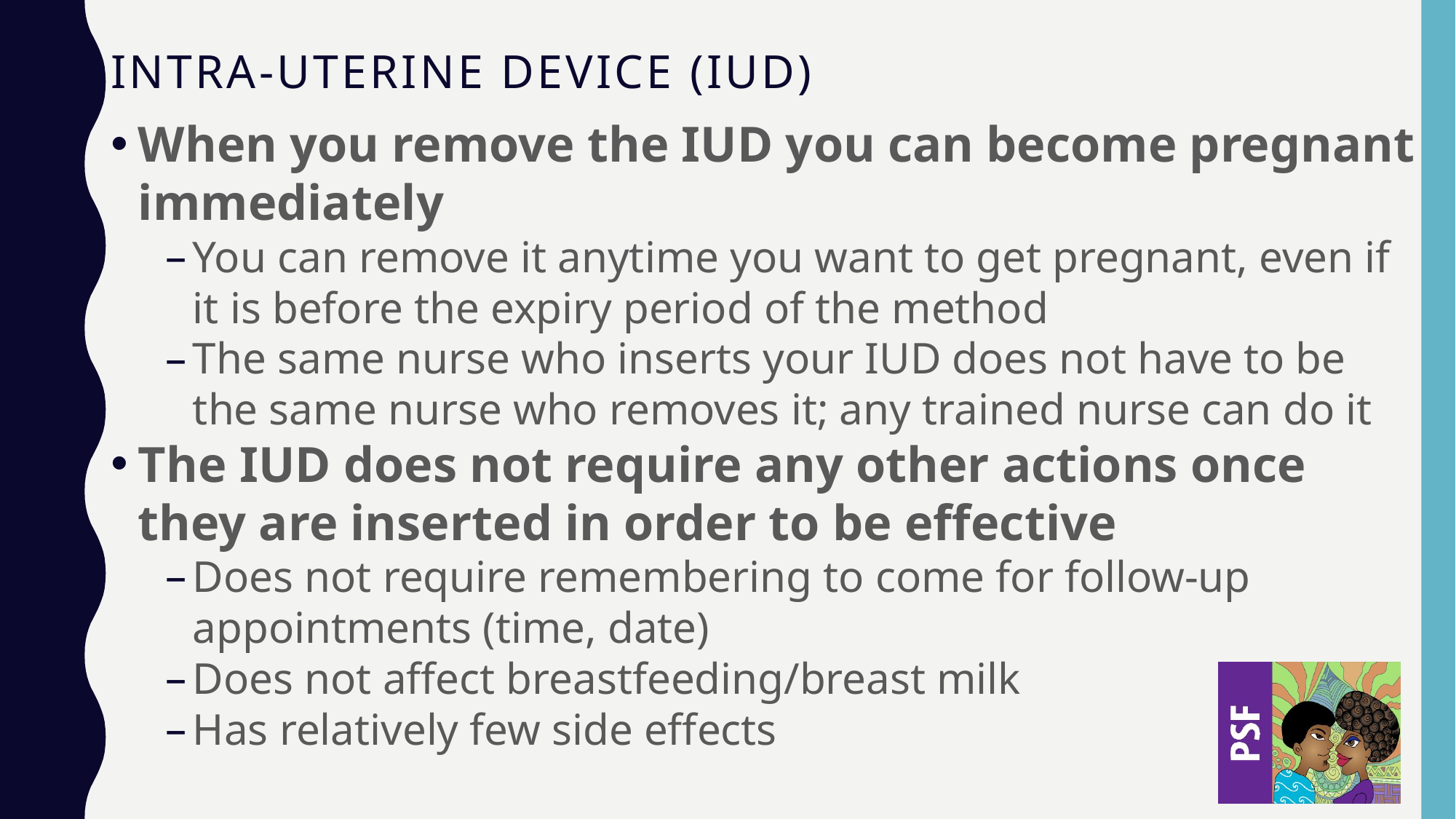

# Intra-Uterine Device (IUD)
When you remove the IUD you can become pregnant immediately
You can remove it anytime you want to get pregnant, even if it is before the expiry period of the method
The same nurse who inserts your IUD does not have to be the same nurse who removes it; any trained nurse can do it
The IUD does not require any other actions once they are inserted in order to be effective
Does not require remembering to come for follow-up appointments (time, date)
Does not affect breastfeeding/breast milk
Has relatively few side effects

## Slide 8
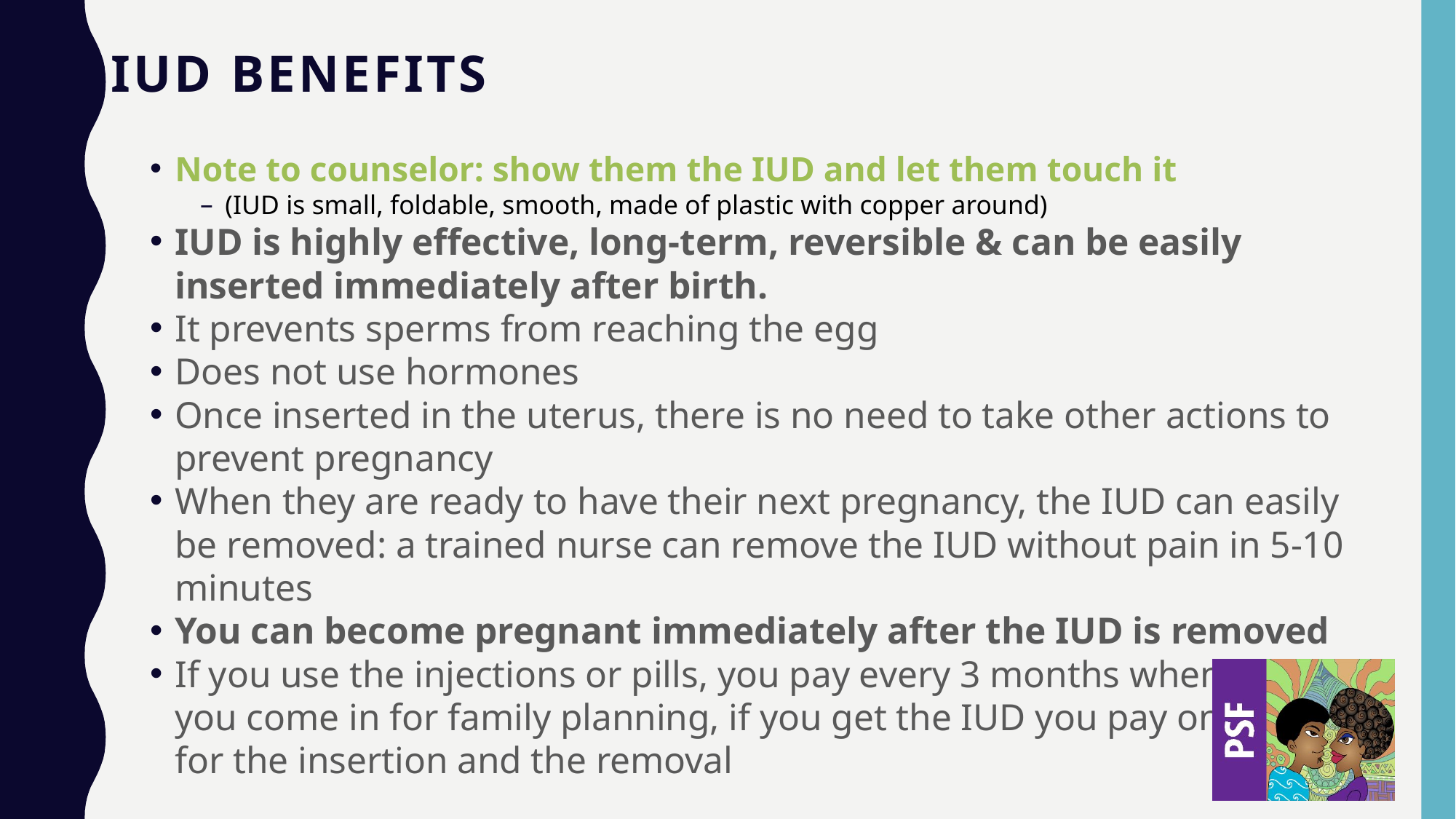

# IUD Benefits
Note to counselor: show them the IUD and let them touch it
(IUD is small, foldable, smooth, made of plastic with copper around)
IUD is highly effective, long-term, reversible & can be easily inserted immediately after birth.
It prevents sperms from reaching the egg
Does not use hormones
Once inserted in the uterus, there is no need to take other actions to prevent pregnancy
When they are ready to have their next pregnancy, the IUD can easily be removed: a trained nurse can remove the IUD without pain in 5-10 minutes
You can become pregnant immediately after the IUD is removed
If you use the injections or pills, you pay every 3 months when you come in for family planning, if you get the IUD you pay only 	 for the insertion and the removal

## Slide 9
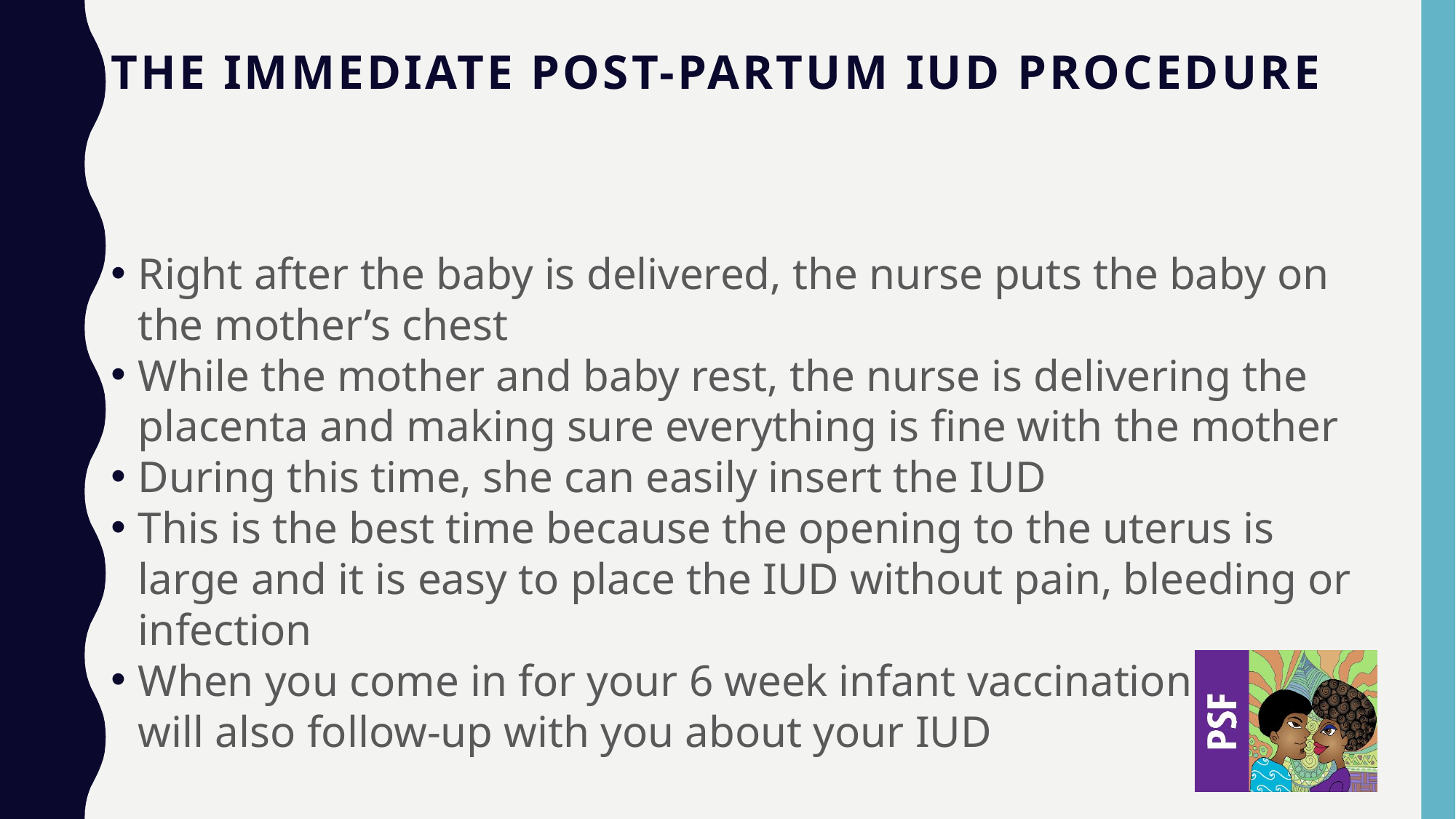

# The immediate post-partum IUD Procedure
Right after the baby is delivered, the nurse puts the baby on the mother’s chest
While the mother and baby rest, the nurse is delivering the placenta and making sure everything is fine with the mother
During this time, she can easily insert the IUD
This is the best time because the opening to the uterus is large and it is easy to place the IUD without pain, bleeding or infection
When you come in for your 6 week infant vaccination visit we will also follow-up with you about your IUD

## Slide 10
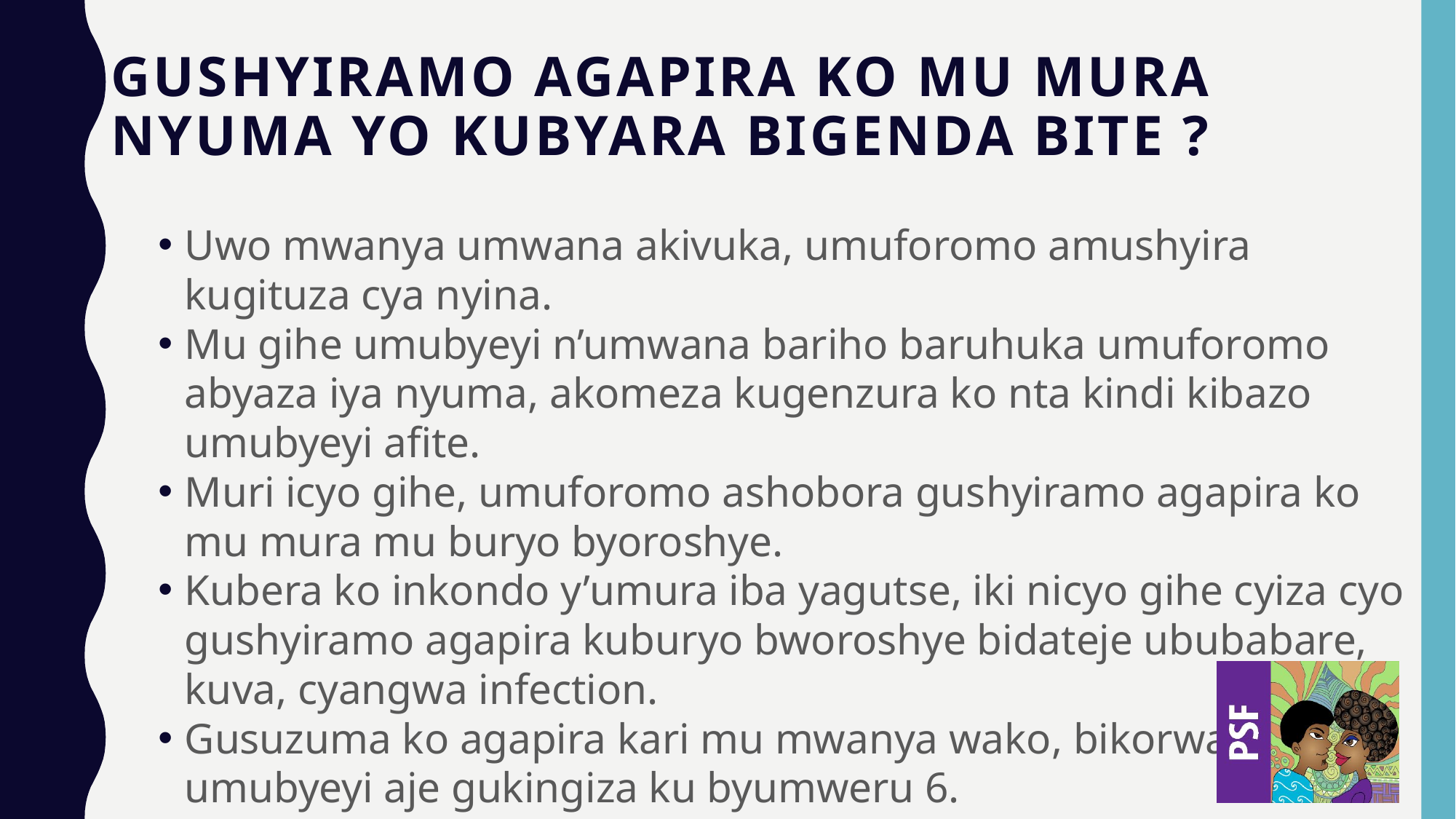

# GUSHYIRAMO AGAPIRA KO MU MURA nyuma yo kubyara BIGENDA bite ?
Uwo mwanya umwana akivuka, umuforomo amushyira kugituza cya nyina.
Mu gihe umubyeyi n’umwana bariho baruhuka umuforomo abyaza iya nyuma, akomeza kugenzura ko nta kindi kibazo umubyeyi afite.
Muri icyo gihe, umuforomo ashobora gushyiramo agapira ko mu mura mu buryo byoroshye.
Kubera ko inkondo y’umura iba yagutse, iki nicyo gihe cyiza cyo gushyiramo agapira kuburyo bworoshye bidateje ububabare, kuva, cyangwa infection.
Gusuzuma ko agapira kari mu mwanya wako, bikorwa igihe umubyeyi aje gukingiza ku byumweru 6.

## Slide 11
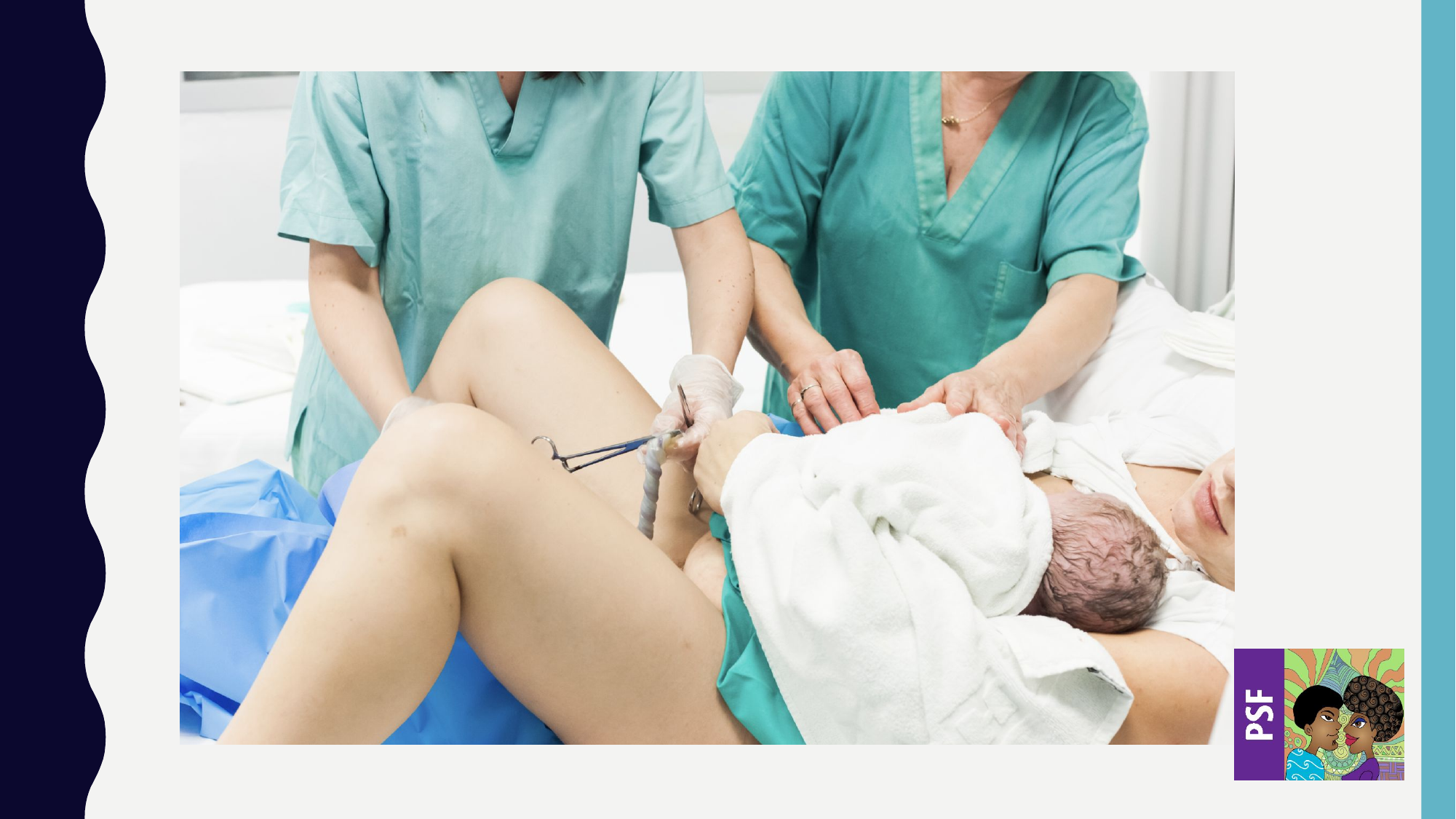

## Slide 12
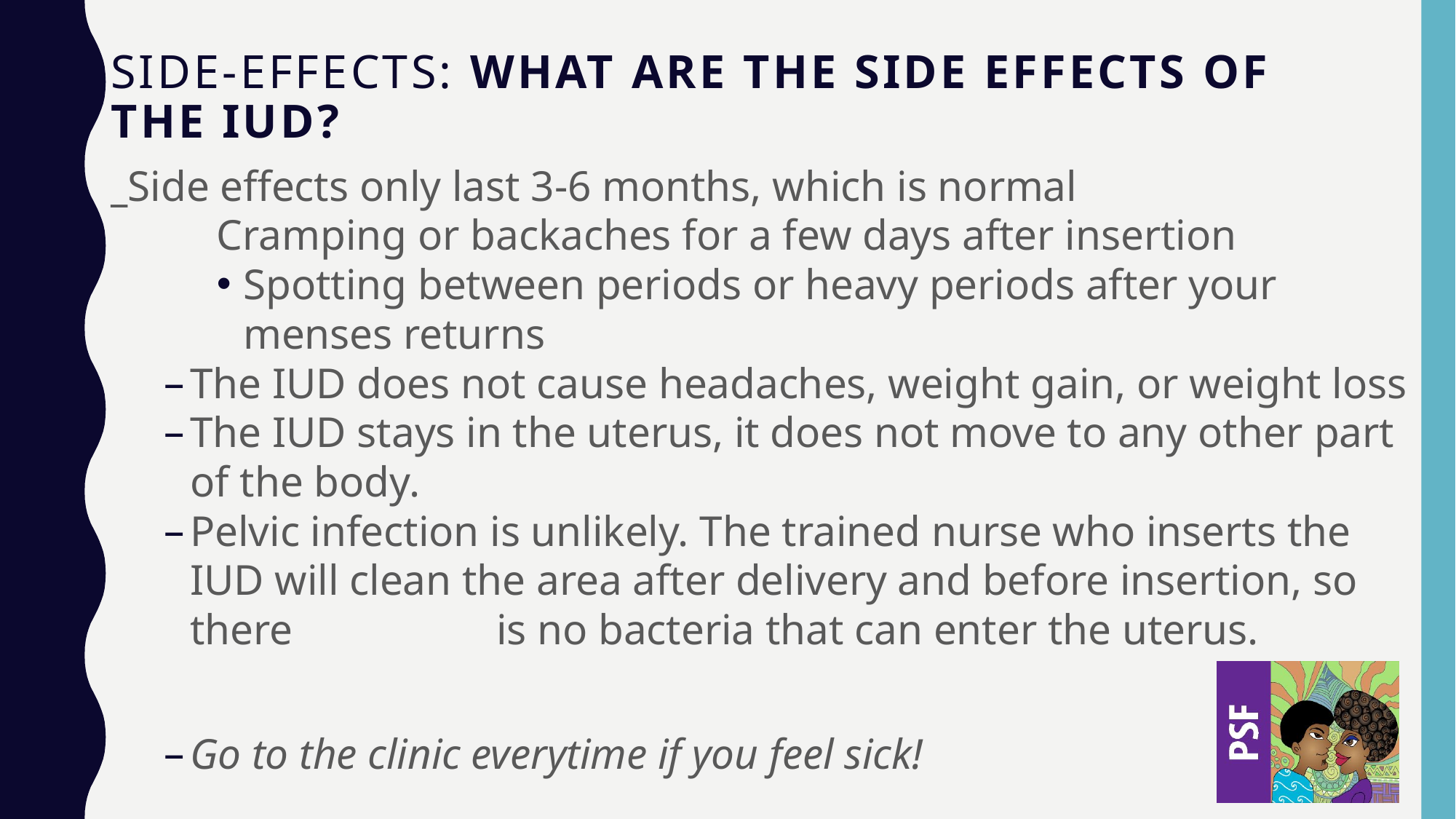

# Side-effects: What are the side effects of the iud?
_Side effects only last 3-6 months, which is normal
Cramping or backaches for a few days after insertion
Spotting between periods or heavy periods after your menses returns
The IUD does not cause headaches, weight gain, or weight loss
The IUD stays in the uterus, it does not move to any other part of the body.
Pelvic infection is unlikely. The trained nurse who inserts the IUD will clean the area after delivery and before insertion, so there is no bacteria that can enter the uterus.
Go to the clinic everytime if you feel sick!

## Slide 13
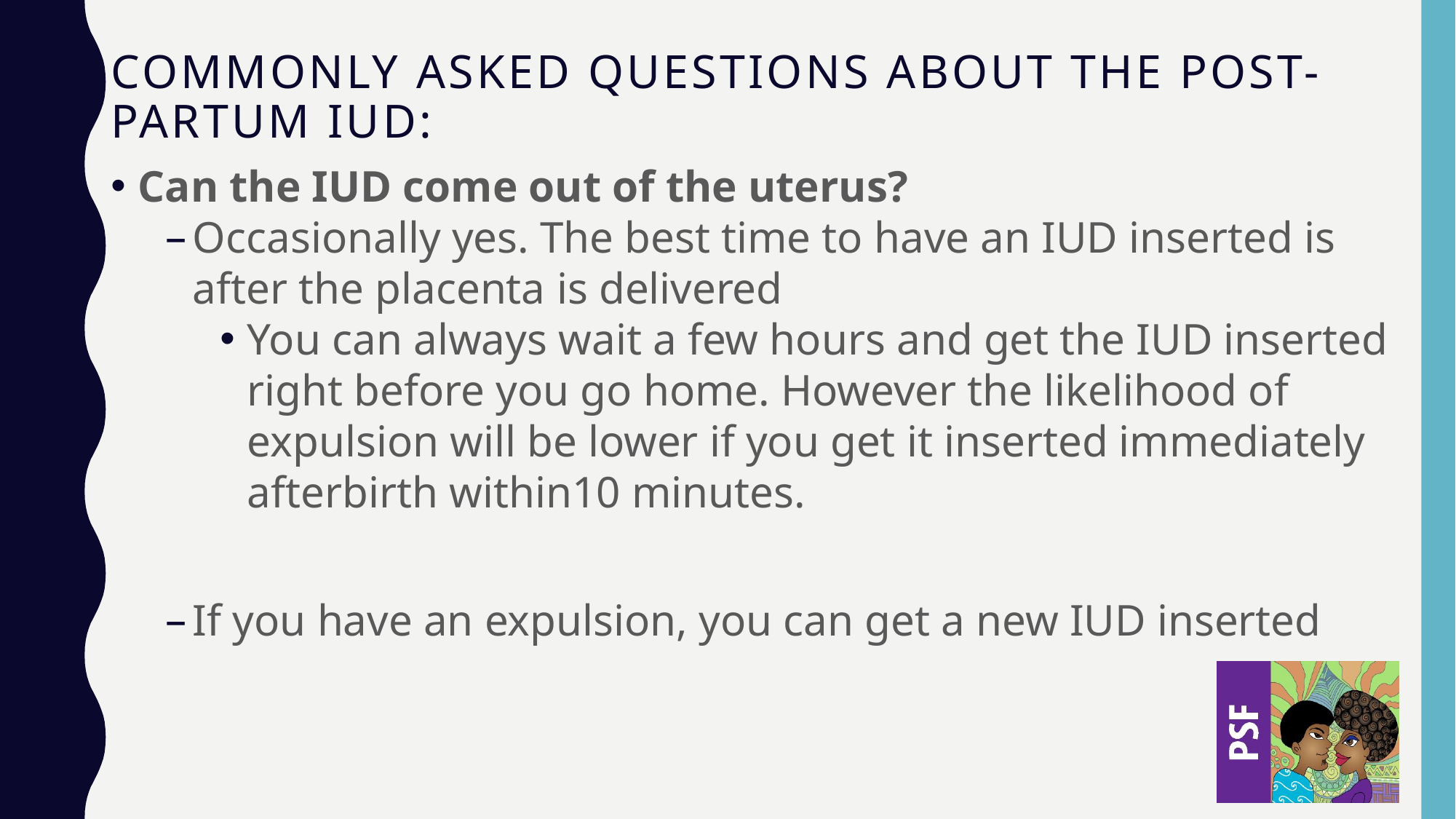

# commonly asked questions about the post-partum IUD:
Can the IUD come out of the uterus?
Occasionally yes. The best time to have an IUD inserted is after the placenta is delivered
You can always wait a few hours and get the IUD inserted right before you go home. However the likelihood of expulsion will be lower if you get it inserted immediately afterbirth within10 minutes.
If you have an expulsion, you can get a new IUD inserted

## Slide 14
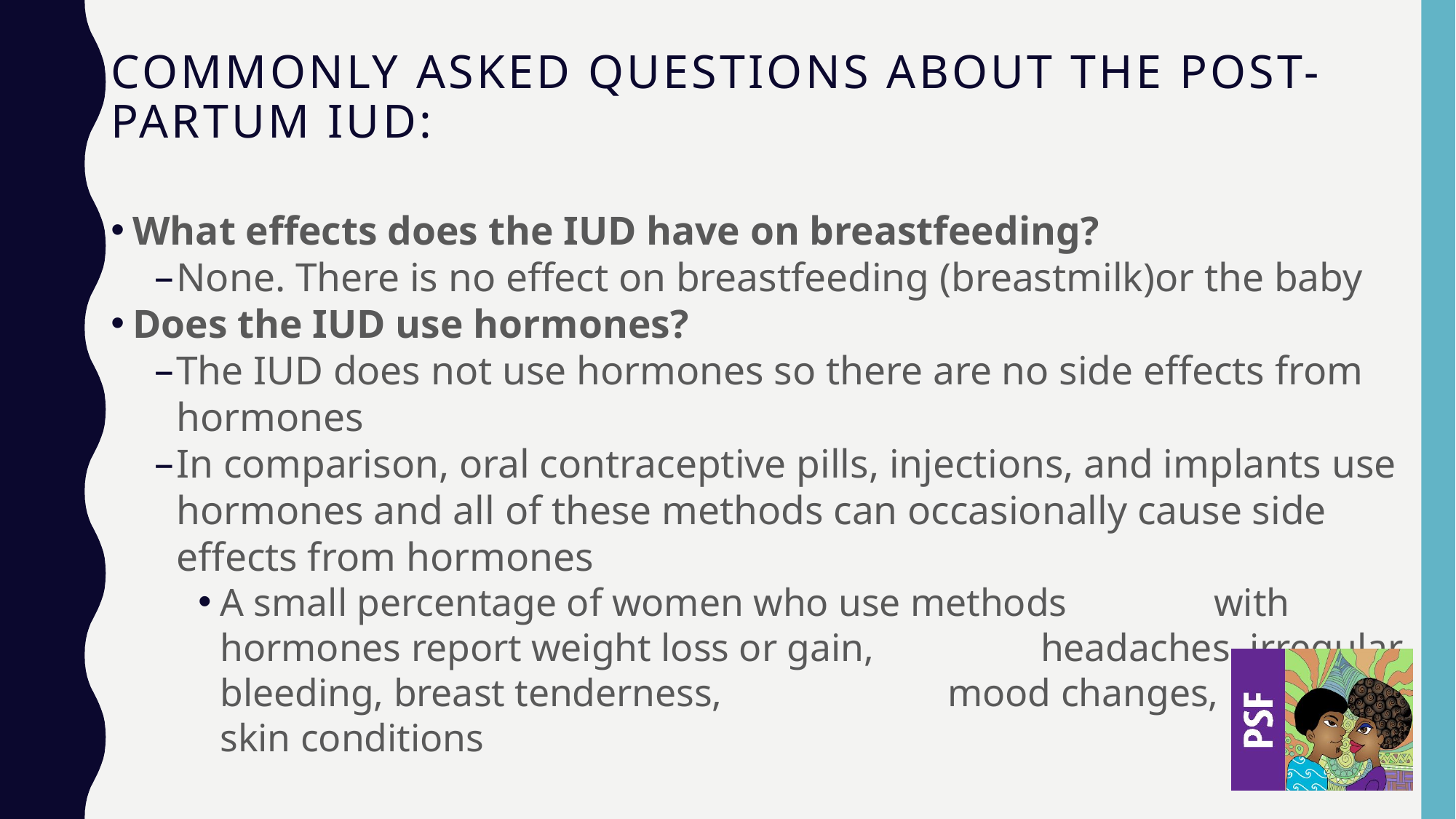

# Commonly asked questions about the post-partum IUD:
What effects does the IUD have on breastfeeding?
None. There is no effect on breastfeeding (breastmilk)or the baby
Does the IUD use hormones?
The IUD does not use hormones so there are no side effects from hormones
In comparison, oral contraceptive pills, injections, and implants use hormones and all of these methods can occasionally cause side effects from hormones
A small percentage of women who use methods with hormones report weight loss or gain, headaches, irregular bleeding, breast tenderness, mood changes, nausea, or skin conditions

## Slide 15
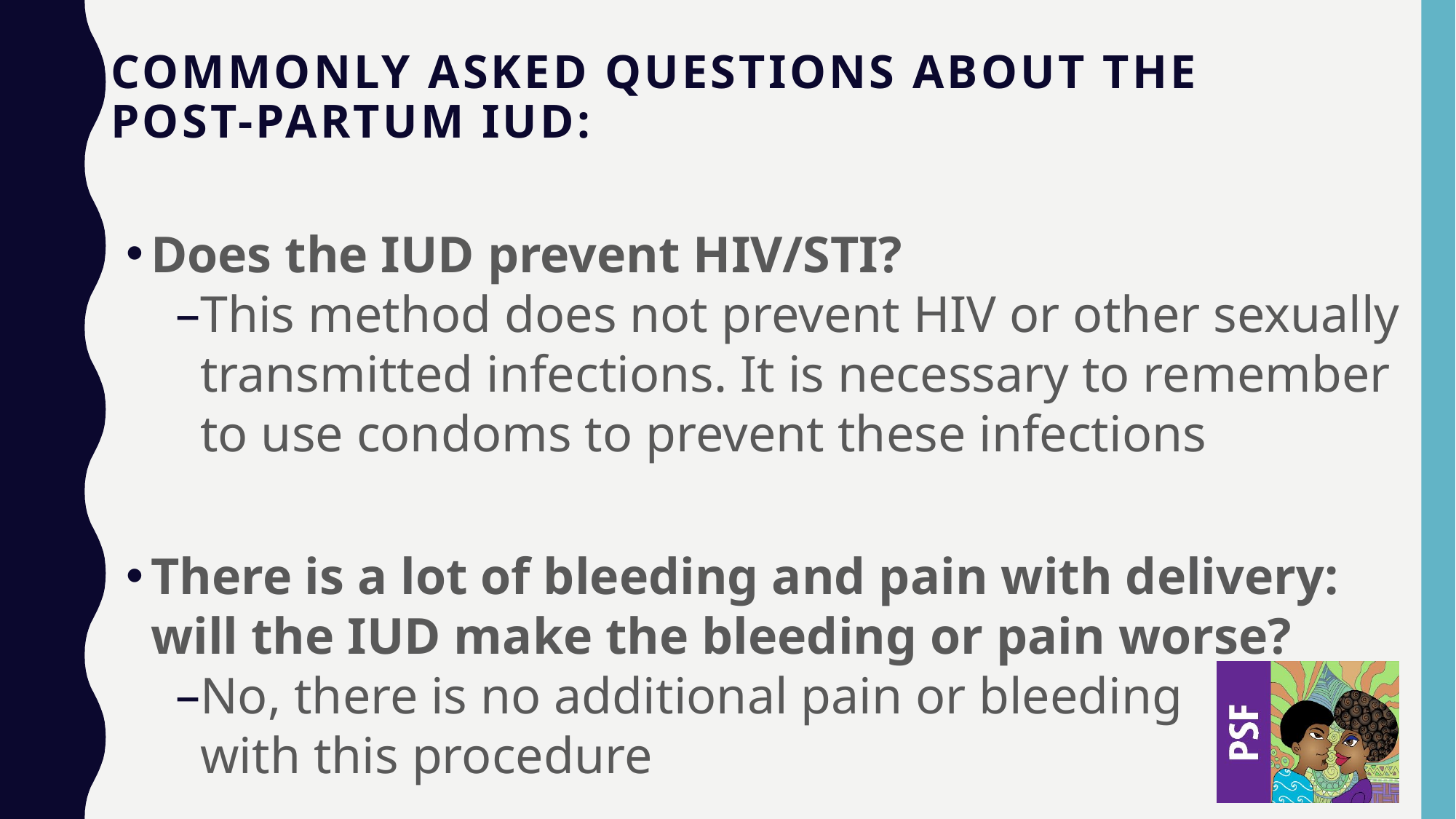

# Commonly asked questions about the post-partum IUD:
Does the IUD prevent HIV/STI?
This method does not prevent HIV or other sexually transmitted infections. It is necessary to remember to use condoms to prevent these infections
There is a lot of bleeding and pain with delivery: will the IUD make the bleeding or pain worse?
No, there is no additional pain or bleeding with this procedure

## Slide 16
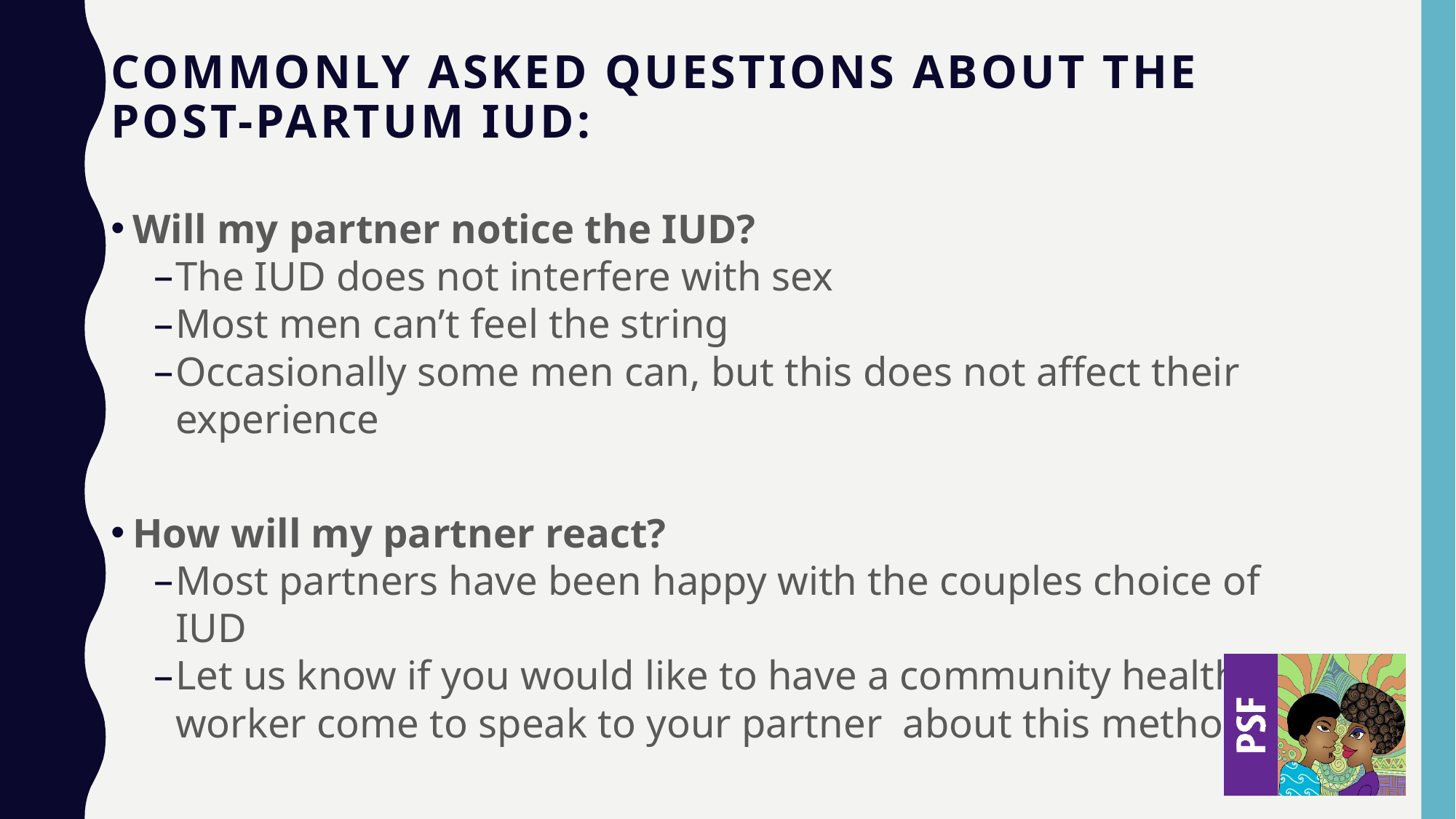

# Commonly asked questions about the post-partum IUD:
Will my partner notice the IUD?
The IUD does not interfere with sex
Most men can’t feel the string
Occasionally some men can, but this does not affect their experience
How will my partner react?
Most partners have been happy with the couples choice of IUD
Let us know if you would like to have a community health worker come to speak to your partner about this method

## Slide 17
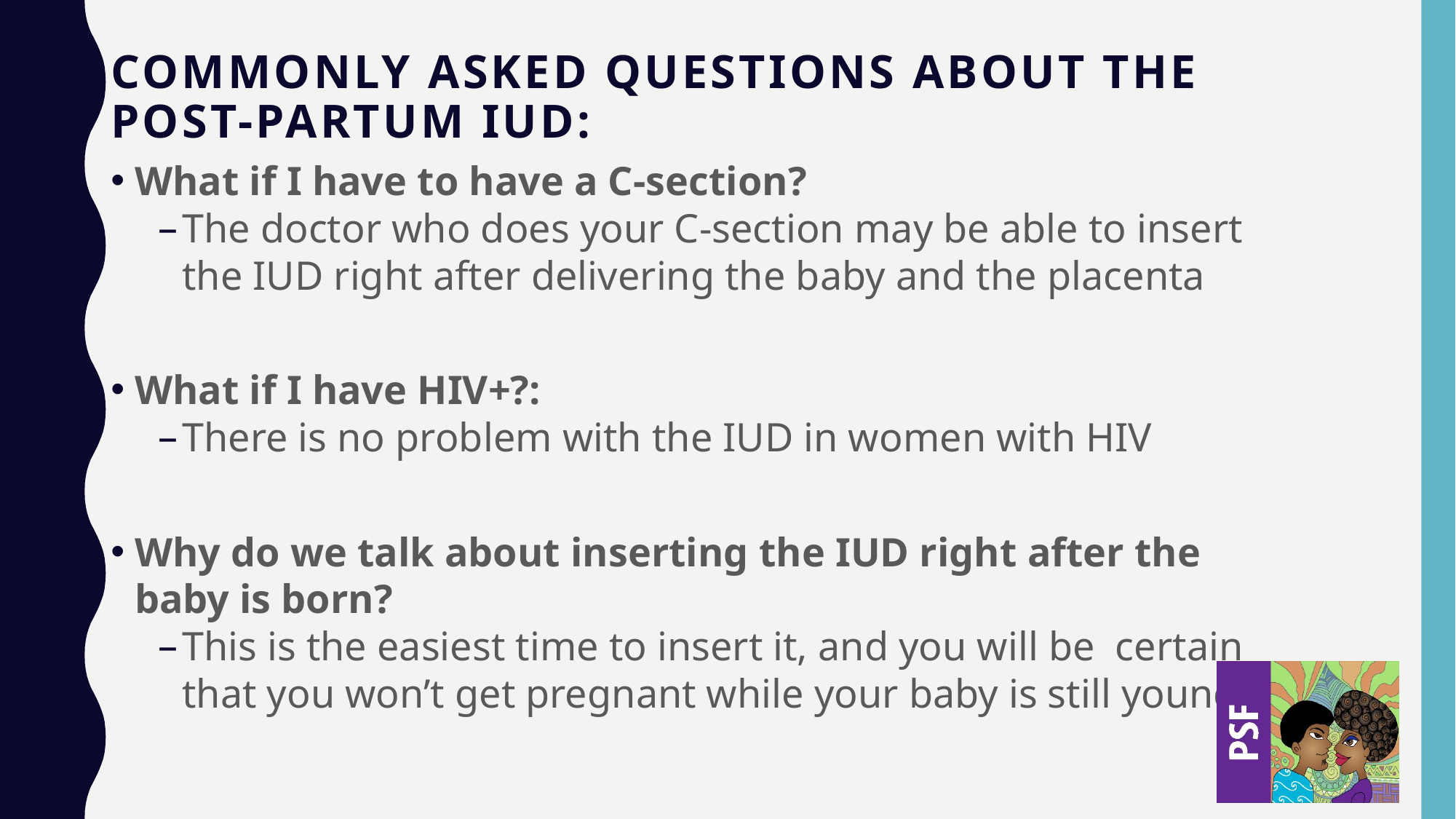

# Commonly asked questions about the post-partum IUD:
What if I have to have a C-section?
The doctor who does your C-section may be able to insert the IUD right after delivering the baby and the placenta
What if I have HIV+?:
There is no problem with the IUD in women with HIV
Why do we talk about inserting the IUD right after the baby is born?
This is the easiest time to insert it, and you will be certain that you won’t get pregnant while your baby is still young

## Slide 18
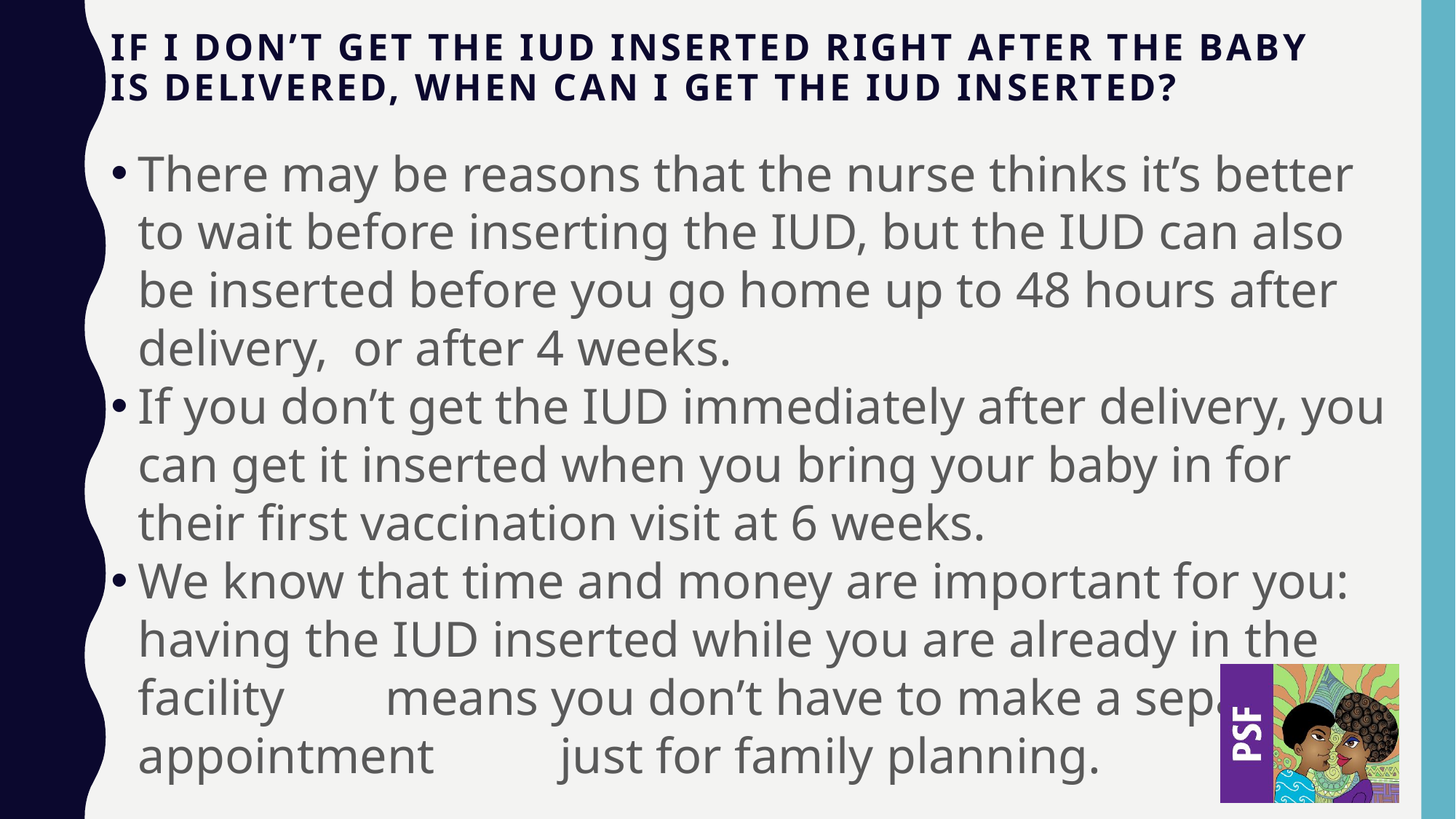

# If I don’t get the IUD inserted right after the baby is delivered, when can I get the iud inserted?
There may be reasons that the nurse thinks it’s better to wait before inserting the IUD, but the IUD can also be inserted before you go home up to 48 hours after delivery, or after 4 weeks.
If you don’t get the IUD immediately after delivery, you can get it inserted when you bring your baby in for their first vaccination visit at 6 weeks.
We know that time and money are important for you: having the IUD inserted while you are already in the facility means you don’t have to make a separate appointment just for family planning.

## Slide 19
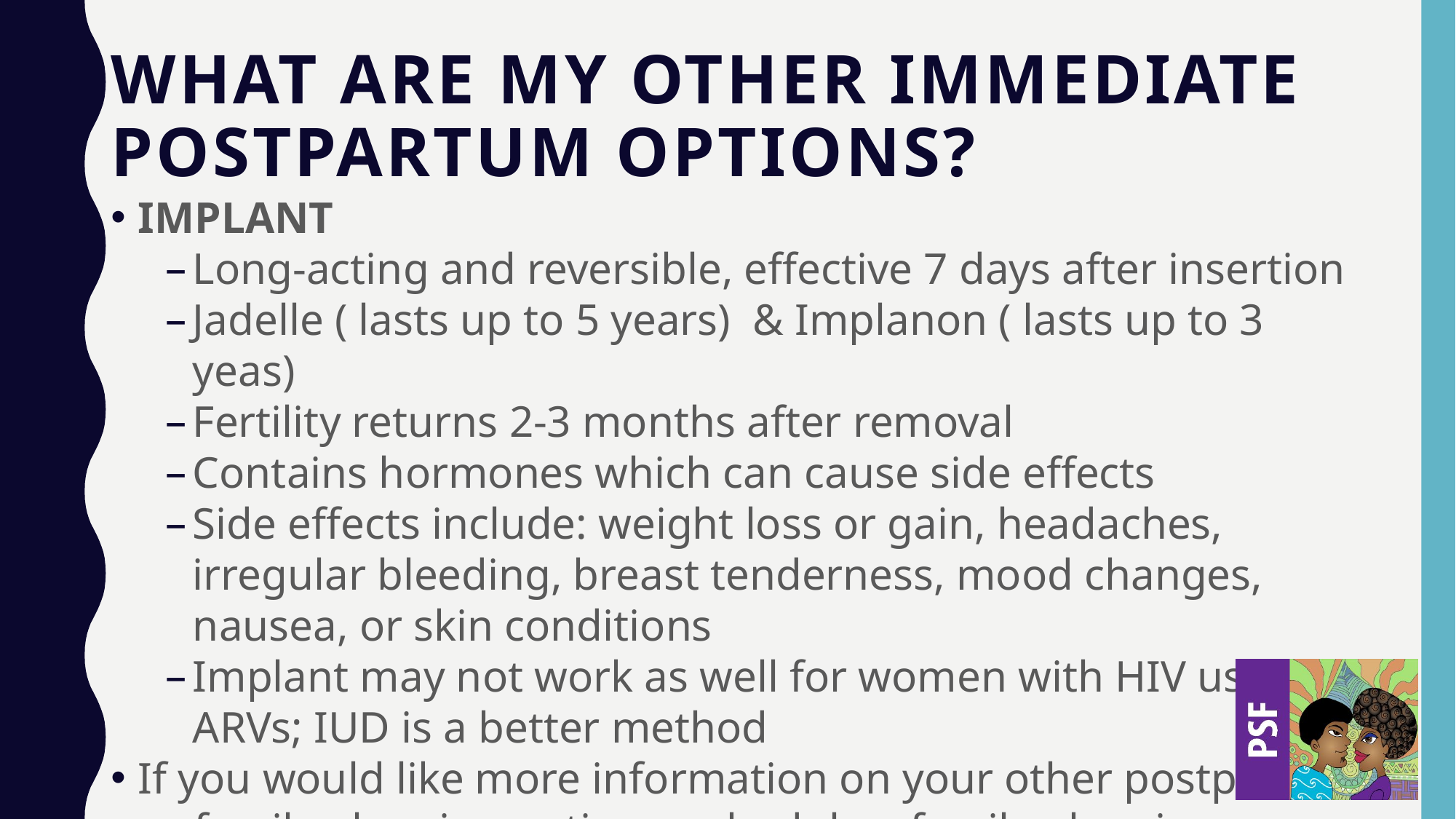

# What are my other immediate postpartum options?
IMPLANT
Long-acting and reversible, effective 7 days after insertion
Jadelle ( lasts up to 5 years) & Implanon ( lasts up to 3 yeas)
Fertility returns 2-3 months after removal
Contains hormones which can cause side effects
Side effects include: weight loss or gain, headaches, irregular bleeding, breast tenderness, mood changes, nausea, or skin conditions
Implant may not work as well for women with HIV using ARVs; IUD is a better method
If you would like more information on your other postpartum family planning options, schedule a family planning consultation

## Slide 20
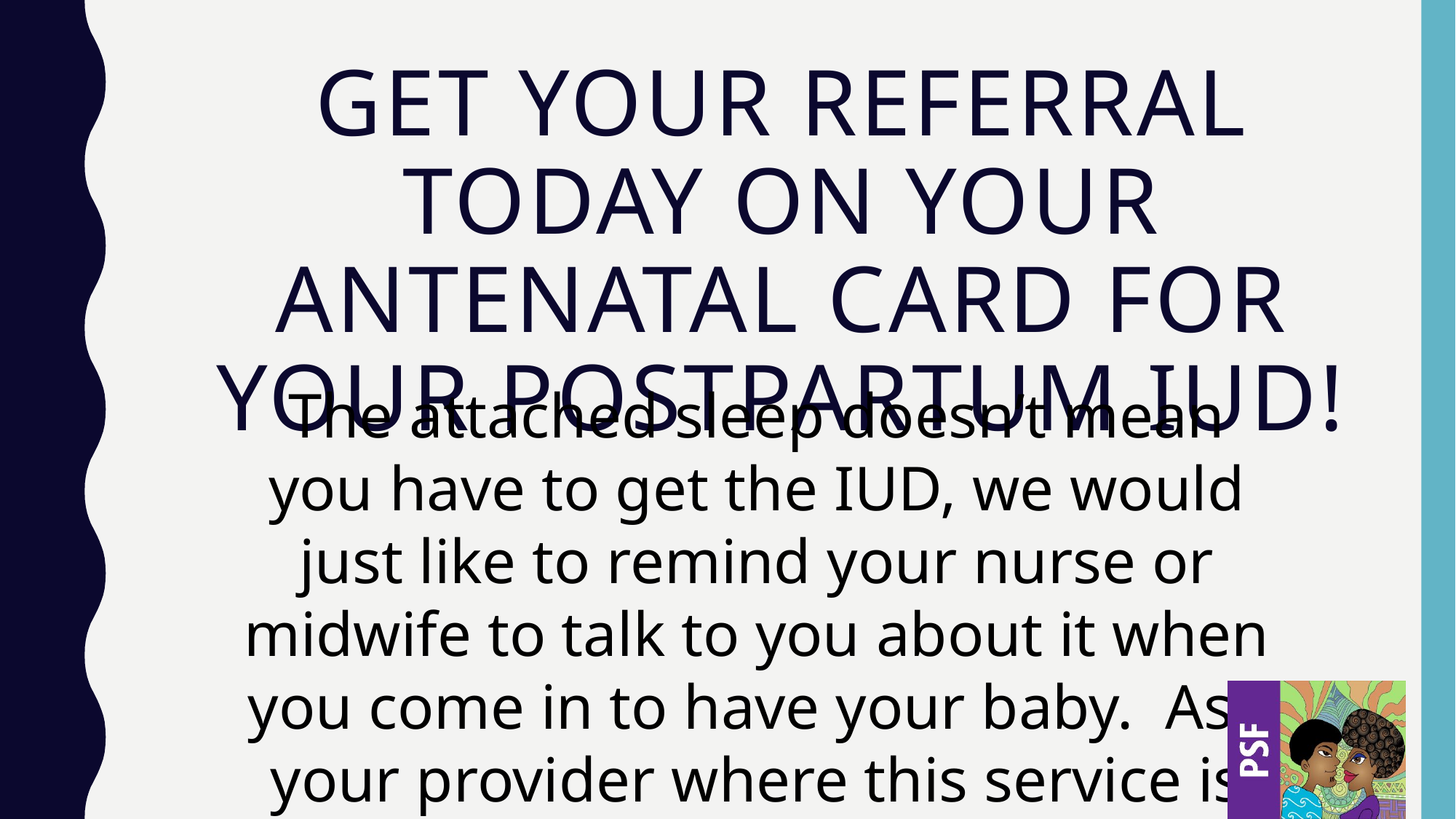

# Get your referral today on your antenatal card for your postpartum IUD!
The attached sleep doesn’t mean you have to get the IUD, we would just like to remind your nurse or midwife to talk to you about it when you come in to have your baby. Ask your provider where this service is available!
