# Supplementary material for: Evaluation of a multi-level intervention to improve postpartum intrauterine device services in Rwanda
Source: Gates Open Res. 2019 Feb 4;2:38. Originally published 2018 Aug 20. [Version 3] doi: 10.12688/gatesopenres.12854.3 (PMC6266741; doi:10.12688/gatesopenres.12854.3)
Supplement: Supplementary file 2 [file gatesopenres-2-14022-s0001.tgz › 7befa5a4-d6d9-4f20-b4cc-64423970f6e8.doc]

**PPDIU EVALUATION PRE TEST	CODE : ----------------------------------**

INSTRUCTIONS : Please indicate if the following statements are **True** or **False**

| Questions | Réponse  **T or F** |
| --- | --- |
| 1. In many developing countries, postpartum women have a better access to family planning services than women who are not postpartum. |  |
| 2. Couples and individuals have right to take their own decision regarding family planning and spacing the next child. |  |
| 3. Women should wait 3 months after a miscarriage before trying to become pregnant again ? |  |
| 4. The expulsion rate is lowest when the IUD is inserted within 10 minutes of delivery of the placenta. |  |
| 5. Placement of an IUD during the immediate postpartum period has a slightly higher risk of uterine perforation than placement during the interval between pregnancies.. |  |
| 6. When the baby is 1 week old , it is an acceptable time to insert a postpartum IUD. |  |
| 7. A woman who breastfeeds can use IUD, implants, injectable, Bilateral tubal ligation as well as Progestin only pills during immediate postpartum period. |  |
| 8. It would be safe, to insert an IUD immediately after delivery of placenta to a woman who has had ruptured membranes for 12 hours. |  |
| 9. The PPIUD should be stitched in place at the fundus with 0 chromic suture when it is placed during cesarean section. |  |
| 10. Inserting IUD manually is the best technique to insert IUD after 48 hours of delivery. |  |
