# Supplementary material for: Evaluation of a multi-level intervention to improve postpartum intrauterine device services in Rwanda
Source: Gates Open Res. 2019 Feb 4;2:38. Originally published 2018 Aug 20. [Version 3] doi: 10.12688/gatesopenres.12854.3 (PMC6266741; doi:10.12688/gatesopenres.12854.3)
Supplement: Peer review history

# Peer review: Reviewer response for version 3

**DOI:** 10.21956/gatesopenres.14022.r26901

**Published:** 2019-02-05

**Authors:** Aurélie Brunie, Trinity Zan

**Affiliations:** 1 FHI 360, Washington, DC, USA; 2 Technical Advisor, Research Utilization, FHI 360, Washington, DC, USA

We believe that the paper could be strengthened by addressing some of the other comments that were not incorporated in this revision, but have nothing to add.

We have read this submission. We believe that we have an appropriate level of expertise to confirm that it is of an acceptable scientific standard.

# Peer review: Reviewer response for version 2

**DOI:** 10.21956/gatesopenres.13955.r26709

**Published:** 2018-11-07

**Authors:** Mark Hathaway, Holly Blanchard

**Affiliations:** 1 Jhpiego, Baltimore, MD, USA; 2 Unity Health Care, Washington, DC, USA; 3 Formerly with Jhpiego, Baltimore, MD, USA

We have read this submission. We believe that we have an appropriate level of expertise to confirm that it is of an acceptable scientific standard.

# Peer review: Reviewer response for version 2

**DOI:** 10.21956/gatesopenres.13955.r26708

**Published:** 2018-11-07

**Authors:** Aurélie Brunie, Trinity Zan

**Affiliations:** 1 FHI 360, Washington, DC, USA; 2 Technical Advisor, Research Utilization, FHI 360, Washington, DC, USA

Thank you to the authors for revising the manuscript. The new version addresses many of the comments we and other reviewers provided. We think that a few things still need to be addressed to clarify the manuscript. 

1. Overall organization/description of Intervention
  We appreciate that the authors have included additional information regarding the intervention design and implementation. However, the organization of the paper is a bit confusing and information related to intervention design and implementation is found elsewhere (as in Results section) and sometimes repeated. We recommend:
  - Clearly adding the dates of the pilot to articulate the design phase (which includes early stakeholder engagement, formative work, and materials development) and the intervention phase (which includes the training and the period of time that services were offered and for which data was reviewed).
  - Labeling a section “Intervention Design”—to include the sections noted in bullet above and which are on p.4 of paper, and the sub-section “Health facility selection and needs assessments” from Results section. It should also incorporate the test in sub-section “PPIUD demand creation” on p.8 which is somewhat repetitive of what is described earlier under “Demand creation”. 
  - Labeling a section for Intervention Implementation—which would include current sections “Postpartum IUD service delivery training” on p.4 through “Reimbursements and other compensation” on p. 6 and would also include the first paragraph under “Data Collection” as this is actually a description of the implementation process, as well as the first paragraph under “Postpartum IUD demand creation and service delivery outcomes” on p.9 as this is also a description of training approach. Move up the sentence on p.6 that starts, “Thus, women and couples could be provided with PPIUD information…” so that this is very clear from the beginning of the description of the intervention.
  - The Results section should begin with the second paragraph in under “Postpartum IUD demand creation and service delivery outcomes.” It should be preceded by a section on data collection and analysis that combines those two sub-sections (and please be clear about whether providers were reporting their own ease AND client anxiety and pain or asking clients to report on anxiety and pain).
  - The last sentence of in the sub-section “Intervention Framework” leads the reader to except an organization that follows Figure 1. Since that is not the case, recommend editing that sentence to indicate that these are woven throughout the description of the Intervention Design and Implementation.
  - When describing the training of PPIUD providers and counselors, it is not very clear that initially providers were trained to counsel only in the last ANC visit, and then it expanded to include L&D providers and (and this part is not clear at all) providers in infant vaccinations? Need to make this clearer under “Implementation Procedures” on p.6.
  - Also, need to be more explicit that “counseling” (or what you call “promotion”—and recall comments from reviewers who recommend against using this term) relies on the use of the counseling flipchart. The description of the counseling on p.9 is a bit confusing since it talks about a special focus PPIUD and PP implant, but also includes counseling on all methods. Please make clearer when you combine these sections.
  - Which CHWs were trained? At one point in time, Rwanda had different types of CHWs, including one type specifically trained to counsel on FP. Were these the ones involved?
2. Figures: We were not able to access Figures 2 and 3 as PowerPoint slides and there are no data points in the pdf file. 
3. Generalizability: We appreciated the explanation of why Kigali was selected and the addition of plans for possible expansion. However, the context for this intervention is quite different from the rest of Rwanda. Please discuss how this impacts generalizability of results in the limitations section.
4. Statistical tests: Technically, statistical tests are not valid since there is no sample that has been taken randomly. They are not really appropriate even if the data may be considered a census (although to be fair, we have it seen this be done by others). 
5. Role of CHWs: The authors mention under demand creation that the insertions and follow-up appointments resulting from the work of CHWs are not shown. Based on this, it is then slightly confusing that details on CHWs are included further in the paper, especially under methods, potentially under Figures 2 and 3 based on legend, and under results, particularly in reference to the increase in insertions. The authors also mention that the effect of the role of CHWs in unclear in the discussion. If the data are not included, how are these observations supported?
6. Male engagement: The authors reference couple counseling under demand creation but then say that a PPIUD counseling occurrence was recorded if women received one-on-one counseling. In the discussion it is mentioned that men were present for 24% of counseling sessions. How does this relate to the one-on-one counseling? Why is this not in the methods and results? What were the outcomes for these clients? More broadly, the scope of the paper is at times confusing because what is presented as part of the intervention is not always aligned with what the paper is then reporting on in terms of methods and results, or the discussion (also see point on CHWs above).  
7. Other contraceptive methods: The authors mention that the flipchart included implants, that providers received increased incentives for both implants and IUDs, and that there is a forthcoming analysis on implant uptake. Based on this and on earlier comments on broader postpartum family planning beyond just IUDs, it is regrettable that implants are not included in this paper. It would be really interesting as a comparison point (as it would be to also see data on what other methods women who were counseled chose, including possibly no method).
8. Discussion:
  - It would be interesting/helpful to offer thoughts on why the majority of insertions shifted from postpartum (10 mins 1 48 hours) in the beginning to post-placental later on. The post placental are discussed in the discussion, but not much is offered as to the shift. The paragraph on bottom of p.11 about low uptake in the 4-6-week window is unclear.
  - The authors posit on p. 11 (“PPIUD demand creation and uptake”) that their intervention contributed to the global body because of the counseling tool developed based on formative work. Would urge a clarification that the formative work is very important, but other PPIUD interventions usually have included a counseling tool, so that is not particularly unique.
  - The discussion on PBF is an important one and linked to the proposed potential sustainability of the intervention. We appreciate the line of inquiry and the argument that compensation should be commensurate with required skill/time. It would be good to at least reference this line of thinking when PBF first described on p.7, perhaps as justification for why PPIUD given twice the normal PBF compensation. Despite efforts to pursue potential increase in PBF or in national insurance compensation, the current incentive doesn’t actually promote sustainability, rather it likely creates a barrier; this should be addressed.

We have read this submission. We believe that we have an appropriate level of expertise to confirm that it is of an acceptable scientific standard, however we have significant reservations, as outlined above.

# Peer review: Reviewer response for version 2

**DOI:** 10.21956/gatesopenres.13955.r26707

**Published:** 2018-10-11

**Authors:** John Cleland

**Affiliations:** 1 Faculty of Epidemiology and Population Health, London School of Hygiene & Tropical Medicine, London, UK

The authors have addressed my concerns and comments. It is still a puzzle why L&D counseling outnumbers ANC counseling by a factor of 3:1 and in the two hospitals the ratio is much higher. In Kacyiru, ANC counseling is negligible. Is this because (a) ANC counseling was not conducted; (b) group counseling elicited virtually no interest; or (c) there were too few staff to conduct one-to-one counseling at ANC clinics?

I have read this submission. I believe that I have an appropriate level of expertise to confirm that it is of an acceptable scientific standard.

# Peer review: Reviewer response for version 1

**DOI:** 10.21956/gatesopenres.13935.r26617

**Published:** 2018-09-28

**Authors:** Mark Hathaway, Holly Blanchard

**Affiliations:** 1 Jhpiego, Baltimore, MD, USA; 2 Unity Health Care, Washington, DC, USA; 3 Formerly with Jhpiego, Baltimore, MD, USA

I agree with the other reviewer's comments and will therefore try to add in a few others rather than repeat.

 The paper adds to other important work in this area showing that an intense, comprehensive, and inclusive intervention strategy can bring about change. Clearer explanation of the steps for the intervention would be helpful.

 One reviewer asks were these facilities and trainees involved in previous PPIUD programs in Rwanda? Several organizations (FHI, Jhpiego, and others) have worked on PPFP and PPIUD programs. It would be good to know this both in terms of sustainability of this project but also how previous work may have fallen short.

 Demand generation utilizing community health workers and performance-based financing may be part of the difference or increased uptake but the author's reflection on this would be very helpful. 

**Definition of PPIUD**: Consider changing the definition of PPIUD to resemble categories 1 &2 in WHO MEC. IUDs are category 2 for postpartum women during the initial 48 hours after expulsion of the placenta. A woman may have an IUD, four weeks after childbirth. This type of insertion technique is completely different than a PPIUD. In light of the fact that the study only had 8% of uptake of PPIUDs during this time (4-6 weeks), we suggest that you drop the results. It is interesting to note that the uptake is low at this time and worth mentioning as an opportune time for FP but clarification is needed.

- Similarly, including the PAC clients, is again a different insertion technique than the PPIUD. Since the numbers were so small, the technique is different, and this is a hot political topic, we believe that adding this data does not serve the post-obstetrical services or clients and suggest that it is dropped. Patients for PAC are often in a different state of mind emotionally as  well, so FP counseling is very different.

**Follow-up: **It is not clear why the study had a 10-day follow-up. The study reported that it used the Jhpiego training that has been developed based on evidence. These training materials advise 6-week follow-up if the client does not have any problems. Frequently the IUD strings have not yet descended since involution of the postpartum uterus is not complete. It would be helpful to explain why the researchers deviated from the 6-week follow-up visit.

- An explanation of what occurred at the follow up is needed. Was it a pelvic exam (bi-manual? speculum??). What is the protocol for a no-string visualized/palpated at time of exam? Is this cost effective to use USG? How will this play out in areas outside of the capital? Again we note that if using the Jhpiego training there is an algorithm for no strings. This may be more generalizabe in settings where no ultrasound is available. Was this a needed deviation from protocol or new protocol?

**PPIUD Promotion: **  We had concerns over the use of the word promotional materials, CHW promoting PPIUDs, and "promoted to". A better explanation of why the unifocal promotion of PPIUD is needed. Did women have a choice on the full range of methods available to them as postpartum women? It would be interesting to know what other methods women chose in the facilities where the study occurred. One may question how ethical it is to “promote” one method. PPFP counseling should be comprehensive to include all available methods. including permanent methods.

- We also had questions about incentivizing the provider with money for the provision of one particular method. This needs to be better explained in the study, how was this overcome, or why the study did not look at this or explain more details.
- Similarly, the CHW piece of the project needs to be better explained—was there an incentive? what are/were the CHW s other tasks? how were they selected to participate in this project? Did they get FP training added onto their other tasks? what was rationale for adding them into the project and how were they financed, i.e., will this be sustained?

**Counseling:** the study developed a flip chart that “promoted” PPIUDs” How was the training rolled out for this? Was this counseling based on any evidence-based counseling techniques such as Balanced Counseling Strategy? The study mentioned that counseling most often occurred during labor. Was this active labor? A clarification about this time and explanation for using this time.

**The intervention** does not seem to be clearly stated. A clarification of the counseling, the cadre of staff inclusive of CHWs, the clinical component; as mentioned earlier PPIUD technique is different from Interval (after 4 weeks) and post-abortion. What was done with the technical working group, were these the stakeholders? If so, who were they or what were their roles, how did the project engage them? 

 We are also confused by the clinical training...was it 2 days with follow up mentoring for 2 weeks? Please clarify.

**Edits:**  In American English, postpartum is one word. Please identify what the initials stand for throughout the study. Consider deleting one of the unpublished studies mentioned in the PPIUD demand creation section. In the organization Jhpiego, only the “J” is capitalized. Remove criticizing other studies in the discussion section. Focus on the result of the study at hand.

We have read this submission. We believe that we have an appropriate level of expertise to confirm that it is of an acceptable scientific standard, however we have significant reservations, as outlined above.

# Peer review: Reviewer response for version 1

**DOI:** 10.21956/gatesopenres.13935.r26616

**Published:** 2018-09-07

**Authors:** Aurélie Brunie, Trinity Zan

**Affiliations:** 1 FHI 360, Washington, DC, USA; 2 Technical Advisor, Research Utilization, FHI 360, Washington, DC, USA

**Is the work clearly and accurately presented and does it cite the current literature?**

 This paper is well-written and structured and cites the existing literature. In general, we congratulate the authors on a very informed, holistic intervention design and a clear presentation of the experience and study.

 Within the 3 ^rd^ paragraph of the Introduction, when referring to women’s attendance at various MCH services, the authors might consider referencing past work in the PPFP community that identifies these “no missed opportunities” and raises challenges with each of those platforms (ANC, L&D, PoNC, vaccination, etc.) - consider looking at PPFP Toolkit on K4Health. In addition, the Programming Strategies for Postpartum Family Planning document from WHO and USAID is an important reference. 

 In the 5 ^th^ paragraph in Intro, the authors nicely articulate the literature regarding barriers to uptake of IUD but could benefit from some time spent also summarizing key challenges providing postpartum women with services, including limited understanding of return to fertility. These are within references cited in the document but suggest pulling them out in a few sentences or a paragraph.

 The intervention framework is clear. As noted by the other reviewer, it would be better to talk about “counseling” rather than “promoted to” in most cases, though it may be related to limited promotion that certain providers offered. However, a clarifying statement could be made and then counseling referred to afterward and throughout. In addition, the authors frequently refer to “supply, demand, and sustainability” but those terms are not featured within the framework. Consider incorporating if possible or referring to them in the explanation of the framework.

 A dedicated emphasis on stakeholder engagement is good to see. However, within the paragraph on early stakeholder engagement, it would be nice to articulate that the FPTWG includes other NGOs and FP implementing organizations. The authors indicated that stakeholders provided “logistical and technical support” through the intervention; it would be helpful to offer some specific examples of how local stakeholders guided the intervention. For example, did government stakeholders participate in selecting participating health facilities (per the following section)? Furthermore, stakeholder engagement during the implementation of the intervention is not clearly described. This is important for replication and points toward sustainability (see comment further down). The authors also mention “champions” a few times but with no description or explanation. This is likely an important element of the design and corresponds to past experiences (including the FHI-supported intervention) and should be included here. These details are helpful for future replication. 

 Under health facility selection, the “procedures” referred to in the last sentence are not very clear - are we referring to family planning operational guidelines?

 In general, the **intervention **is described well and was thoughtfully designed, but could benefit from addressing a few gaps and organizational considerations. Overall, it is important to link the design of this intervention to past work that has been done globally and, especially, in the country. Past efforts in Rwanda (including those supported by us at FHI 360) were cited but more should be said about how this intervention built upon and/or differed from previous efforts. For example, were the selected facilities for this study also involved in past studies? As noted earlier, while the authors refer several times to “supply, demand and sustainability,” it is not clear what components are linked to sustainability. It would be helpful to more clearly articulate this. In addition, it would be interesting to provide a justification for why the authors chose to include the 4-6-week period for PPIUD insertions rather than focus on the first 48 hours only.

 Regarding the **supply side**: Several studies have shown that the supply-side component of PPIUD services is feasible in LMICs, so what, if anything, differed here? It would seem that the training period was shorter, so that may be something to highlight. Was the number of supervised insertions required for certification fewer than in the past? The FHI-supported intervention made use of a stamp on ANC cards to indicate method choice if a woman selected a method during ANC counseling. The authors refer on p.6 (under data collection, which appears to be misplaced) to having a method recorded, but don’t indicate how. Also related to supply (though perhaps it should also be tagged as related to sustainability), the discussion of PBF includes specific dollar figures. It would be more helpful to include how the $57 per month was calculated, and how the amounts cited compare to standard PBF-payments for FP. 

 Past experiences have also shown that it is often the **demand-side** that requires significant attention. The authors likely have more to contribute here, given the formative research that was conducted, however the results of this are not shared, nor linked to how they impact the intervention design. So again, what is new/different here and what builds on past efforts? Did the study team refer to past promotional materials (such as those available from the FHI-supported study)? There are several mentions of male involvement, and the fact that men often attend ANC sessions. In the FHI-supported intervention, we specifically learned to encourage providers to incorporate the PPFP counseling into the first ANC visit, when more men attend. Did you have any similar learnings or lessons to share regarding how men are involved in PPFP decision-making and how the intervention was designed to address that? Given that you also refer to male engagement in the discussion, more details would be helpful. The role of CHWs seems to be a notable difference from past interventions, and one the authors felt important, so more information regarding the decision to include this would be helpful.

**Is the study design appropriate and is the work technically sound?**

 The paper discusses the intervention framework and model, and a compilation of service statistics is used to describe outputs and proximal outcomes. The work presented in this paper does not involve primary data collection.

 Two hospitals and four health centers with high labor and delivery volume in Kigali were selected for the intervention. The authors should provide a rationale for the number of sites selected and the decision to focus only on Kigali, which is likely to offer a different context compared to other regions of Rwanda. It would also be helpful to note (if known) whether the participating sites had previously participated in PPIUD interventions.

 The paper references formative work including focus group discussions and surveys. However, these were conducted to inform some of the intervention components and are not part of the results presented in this paper. Therefore, the statement on ethical considerations and consent does not seem relevant. The discussion on p. 7 of PPIUD demand creation should shift to earlier, as part of the description of the intervention itself and more detail on results added (per an earlier comment). The authors should provide information pertaining to the methods and results presented in this paper. As a note, later in the paper under methods, information on the duration of FGDs and surveys and on compensation similarly does not seem relevant.

 As mentioned by the authors, this is a pre-post design with no control group, which carries limitations, although the authors offer a justification by saying that there were no other interventions or changes over the study period and the striking change is convincing. However, findings related to uptake over time still need to be interpreted cautiously due to other possible underlying trends and to changes in the intervention model (e.g. addition of promotion by CHWs and introduction of PBF).

 One barrier identified in the introduction is male involvement, and the intervention mentions promotion to couples. Are there any data on promotion to couples that can be included? This seems to be a potential gap in the paper which otherwise seems to address many of the barriers identified in the background.

 The focus of the intervention is on PPIUD. However, some components – notably around demand-generation – address PPFP more broadly, which makes sense in the context of choice. Were any data captured in government logbooks that could provide insight into the possibly larger contribution of this intervention to PPFP beyond the PPIUD? The paper would also be strengthened by providing additional data that may put some of the service statistics in perspective. For example, are there any data on the total number of women attending the different services that could be cited to allow the reader to gauge the proportion that was reached through promotional activities? Or are there any data on the total number of institutional deliveries that could be provided for context?

**Are sufficient details of methods and analysis provided to allow replication by others?**

 The methods in the abstract should cover data collection and not only the intervention model. Some of the results (about the six months prior to the intervention) are not clearly mentioned in the paper itself.

 Regarding the methods section, the authors should cross-reference the results section with the methods (in particular data collection) section to make sure that all relevant information is included. For example, the paper would benefit from additional information regarding: selection criteria for providers trained in PPIUD service delivery (was being a woman a selection criterion? Why were 85% of providers from L&D and only 15% from FP? Did providers have prior experience with PPIUD insertion given the national curricula?); how information on client age and parity was obtained; who collected information on provider ease, client anxiety, and client pain (and could this be a source of bias?) and on what tool; how information on follow-up outcomes was documented. The data collection section mentions that data was recorded in government logbooks; however, there seems to be more information that is typically routinely collected in many countries or can easily fit in one logbook. Collecting and extracting service statistics can be challenging and time-consuming, and others may be interested in how so much information was collected for replication. Were registers adapted? Were other tools used? Was the study team able to link all data with unique identifiers (as mentioned) or were there missing data?

 Please note that the reference to the two unpublished manuscripts under PPIUD demand creation development may not be placed in the right sentence (the sentence mentions CHWs and providers, but the papers seem to be based on data collected with women and couples).

 The paper offers a description of most of the analyses, but a few details are missing. The authors should for example describe the tests used to assess the association between timing of promotion and uptake and consider whether a statistical test is justified. Results on promotion do not seem to include promotion by CHWs. One challenge is that this component was only included later. However, some information on its contribution would be interesting. The authors should also clarify how promotions were counted when the same person was reached more than once. Were they excluded from Table 1 or counted based on the first contact, for example?

**If applicable, is the statistical analysis and its interpretation appropriate?**

 The analyses are descriptive and cover the different aspects of the intervention. In some cases, the authors may want to add some information to provide the reader with more insight. Some examples are: breakdown of the 83 providers trained to promote PPIUD by CHWs vs. facility (and by FP, ANC, L&D, IZ); more information on why 10% of providers trained in PPIUD service delivery were not certified (and possibly data on pre- and post-test scores); and women promoted to by CHWs. Additionally, more text that expands on the data presented in the tables, particularly when variation across sites exists, would be helpful. It would also be helpful to spend time in the discussion offering hypotheses for why the type of insertion changed over time and differs from other studies. In particular, the low level of intra-cesarean is notable, especially since the two large hospitals likely do a fair number of cesareans. The authors offer references to show the comparison with other countries but don’t offer ideas for why. Much of the gray literature spends time discussing the challenges with training providers to offer the different types of insertion, their comfort level, etc. - this would be an interesting discussion to include here as well.

 Figure 2 shows PPIU insertions over time, and the insertion of arrows showing the timing of different events is useful. There is a very clear increase at the beginning of the intervention; however, conclusions regarding the possible effect of adding PBF-incentives or promotion by CHWs (beyond an initial spike) are complicated by the few data points available and staffing changes. In addition, since both PBF-incentives and CHW promotion were added at the same time, it is difficult to separate their possible contributions. More broadly, a few months after each intervention component, the number of insertions seems to decline. This raises questions regarding sustainability (although levels still seem to be above the baseline). It would be interesting to offer potential hypotheses explaining this.

**Are all the source data underlying the results available to ensure full reproducibility?**

 The authors submitted a dataset and codebook that seems to include variables related to insertion and follow-up outcomes. The codebook mentions a variable which is a count of promotion contacts but does not seem to include information on type of contacts. Variables related to test scores of providers during training are not provided but are currently not discussed in the paper.

**Are the conclusions drawn adequately supported by the results?**

 A lot was accomplished through this project, from intervention development through implementation, reaching many women and inserting thousands of PPIUDs, which is note-worthy given historical challenges with IUD uptake in general, and the service delivery challengers with PPIUD. The conclusions are largely supported by the results and the discussion also draws on other studies for additional context. However, given the design, the authors may want to avoid the word “significantly” in the conclusion. Another area that may benefit from some clarification or rewording is the section on supply. Earlier work in Rwanda already demonstrated that PPIUD is feasible, and the authors should clarify in what way this project adds to earlier evidence. In addition, the authors note there was some (spontaneous?) training and mentoring and highlight this as a sign of sustainability. Additional information on the quality of services provided by the newly trained staff (given the earlier focus on pre- and post-testing and certification) may be useful to support this statement. Likewise, the authors should elaborate as to why PBF-incentives appeared to support providers and facilities, and how this affects sustainability. Overall, given earlier work on PPIUD in Rwanda leading to the development of a national curriculum and reporting mechanisms but subsequently low levels of uptake (including at one of the facility which was both included in the earlier work and in this study), sustainability is an important question. As noted previously, we would encourage the authors to spend a bit more time describing what components of the intervention were hypothesized to contribute to the sustainability approach, and what evidence supports whether those components were effective. These likely include the stakeholder engagement, champions, cost (it is nice that cost information is included), as well as the extent to which this built on local capacity and past experiences.  It would be important to link this study to others in the past, drawing conclusions about what supports sustainable PPIUD services in Rwanda, what else needs to be in place, and what might we expect to see in the future.

We have read this submission. We believe that we have an appropriate level of expertise to confirm that it is of an acceptable scientific standard, however we have significant reservations, as outlined above.

# Peer review: Reviewer response for version 1

**DOI:** 10.21956/gatesopenres.13935.r26638

**Published:** 2018-09-03

**Authors:** John Cleland

**Affiliations:** 1 Faculty of Epidemiology and Population Health, London School of Hygiene & Tropical Medicine, London, UK

One lesson from this paper is that, when change is large and abrupt in the presence of an intervention, a simple before-and- after study is as convincing as a randomized control trial with regards to causal attribution. In the 6 months prior to the intervention only 46 PPIUDs had been inserted. In the 12 months following, the total rose to 2633 at the four study facilities. Clearly, the intervention had a large impact. Moreover, follow-up data indicate a low level of adverse events, such as expulsions, removals and infection. This an important addition to the sparse literature on PPIUDs in low income countries though some aspects of the presentation need improvement.

 The single most remarkable result is that 29% of the 9063 women counseled on IUDs and who delivered at one of the facilities had an insertion, the majority of which were post-placental and over 90% before discharge. Previous similar multi-country PPIUD interventions recorded acceptance rates of 2-7% among counseled women (Pfitzer et al. and Pleah et al., both cited in the paper). In view of this huge difference, suspicions are raised about the validity of the estimates of numbers of women counseled. And indeed there are surprising elements in the data on counselling. If I have understood table 1 and the text correctly, 95% of women were counseled only once: 65% in the labour/delivery ward, 20% at ANC, 10% at immunization clinics and 6% postpartum. Given the involvement of community health workers in IUD promotion, this distribution of the timing of counselling is strange and rather disturbing. Can women in the trauma of delivery give fully informed consent? In a busy labour ward, how can accurate records be kept of who does and does not receive counselling? Why were so few women counseled more than once? Surely many of the 1786 women counseled at ANC were also counseled in the L&D ward or was some mechanism in place to avoid duplicated counselling, which could explain the low level (9%) of uptake among those counseled at ANC. The paper would be strengthened if these issues were fully discussed. It would also be helpful to know the total number of deliveries at the 4 facilities during August 2017-July 2018 to place the results in a broader context.

 I wonder what role performance-based financing (PBF) played in the success of the intervention. The authors write that a flat sum is routinely paid for FP provision to a new user, regardless of the method. How much is that routine sum? Was the new payment of $1.20 to providers for each insertion instead of the routine sum or in addition to it? How does $1.20 compare to the salaries paid to nurses and midwives?

 Two small points: Why use the awkward phrase “promoted to” instead of counseling? The bottom row of table 2 should be labelled “dissatisfied” not satisfied.

I have read this submission. I believe that I have an appropriate level of expertise to confirm that it is of an acceptable scientific standard, however I have significant reservations, as outlined above.
